# Effect of serum sample storage temperature on metabolomic and proteomic biomarkers

**DOI:** 10.1038/s41598-022-08429-0

**Published:** 2022-03-17

**Authors:** Erkka Valo, Marco Colombo, Niina Sandholm, Stuart J. McGurnaghan, Luke A. K. Blackbourn, David B. Dunger, Paul M. McKeigue, Carol Forsblom, Per-Henrik Groop, Helen M. Colhoun, Charles Turner, R. Neil Dalton

**Affiliations:** 1 Folkhälsan Institute of Genetics, Folkhälsan Research Center, 00290 Helsinki, Finland; 2 Department of Nephrology, University of Helsinki and Helsinki University Hospital, 00290 Helsinki, Finland; 3 Research Program for Clinical and Molecular Metabolism, Faculty of Medicine, University of Helsinki, 00290 Helsinki, Finland; 4 The Usher Institute, University of Edinburgh, Edinburgh, UK; 5 Institute of Genetics and Cancer, University of Edinburgh, Edinburgh, UK; 6 Department of Paediatrics, University of Cambridge, Cambridge, UK; 7 Wellcome Trust-MRC Institute of Metabolic Science, University of Cambridge, Cambridge, UK; 8 Department of Diabetes, Central Clinical School, Monash University, Melbourne, VIC Australia; 9 Department of Public Health, NHS Fife, Kirkcaldy, UK; 10 WellChild Laboratory, Evelina London Children’s Hospital, Guy’s and St Thomas’ National Health Service Foundation Trust, London, UK

**Keywords:** Metabolomics, Proteomics, Biomarkers

## Abstract

Prospective biomarker studies can be used to identify biomarkers predictive of disease onset. However, if serum biomarkers are measured years after their collection, the storage conditions might affect analyte concentrations. Few data exists concerning which metabolites and proteins are affected by storage at − 20 °C vs − 80 °C. Our objectives were to document analytes affected by storage of serum samples at − 20 °C vs − 80 °C, and to identify those indicative of the storage temperature. We utilized liquid chromatography tandem mass spectrometry and Luminex to quantify 300 analytes from serum samples of 16 Finnish individuals with type 1 diabetes, with split-aliquot samples stored at − 80 °C and − 20 °C for a median of 4.2 years. Results were validated in 315 Finnish and 916 Scottish individuals with type 1 diabetes, stored at − 20 °C and at − 80 °C, respectively. After quality control, we analysed 193 metabolites and proteins of which 120 were apparently unaffected and 15 clearly susceptible to storage at − 20 °C vs − 80 °C. Further, we identified serum glutamate/glutamine ratio greater than 0.20 as a biomarker of storage at − 20 °C vs − 80 °C. The results provide a catalogue of analytes unaffected and affected by storage at − 20 °C vs − 80 °C and biomarkers indicative of sub-optimal storage.

## Introduction

Many chronic diseases, such as diabetic complications, are slowly progressive by their nature. In order to target preventive treatment efforts, it is clinically important to be able to identify individuals at the highest risk of complications. Along with clinical risk factors, robust biomarkers are routinely utilized to assess the risk of disease, e.g. among individuals with diabetes, estimated glomerular filtration rate (eGFR) and/or urinary albumin excretion rate (AER) can be measured regularly to assess the risk of developing diabetic kidney disease and end stage renal disease (ESRD)^1^. Identifying individuals at high risk of disease enables early therapeutic intervention and, moreover, the same risk assessment minimizes unnecessary therapeutic intervention and brings clinical reassurance for individuals at low risk. In addition, new therapeutics can be cost-effectively assessed in high-risk individuals alone.

Identifying early prognostic biomarkers in slowly progressing diseases requires collecting and storing biological samples.The samples need to be collected during the natural history of the development of disease complications both cross-sectionally and longitudinally. Ideally, in order to identify early prognostic biomarkers, samples would be collected within the first years of diagnosis of diabetes to be analysed many years later when the complication has developed.

Current best practices for optimal collection and storage of plasma/serum samples include splitting samples into multiple aliquots for single use to avoid multiple freeze–thaw cycles and storing them at − 80 °C ^2^. However, historically, many potentially highly informative cohort samples were stored at − 20 °C and subjected to multiple freeze–thaw cycles. This might inadvertently lead to including low-quality samples, or, excluding valuable samples in biomarker discovery and validation studies due to excessive caution, resulting in failure to identify potential biomarkers or limiting the use of a particular bio-resource.

Many pre-analytical factors, i.e., those processes before sample measurement, potentially affect metabolomic and proteomic studies in serum samples. These include study design, sample collection, sample handling and storage, and sample preparation^3,4^. For example, up to 3% of the detected metabolome was affected by multiple freeze–thaw cycles or/and extended thawing at 4 °C^5^. Surprisingly little data are found on the effect of temperature during long-term storage of serum samples, and more specifically, on the effect of storage at − 20 °C vs − 80 °C. To the best of our knowledge only studies investigating short-term storage are available^6,7^.

The objectives of this study were two-fold: in serum samples collected from subjects with type 1 diabetes, first, to identify biomarkers of storage at − 20 °C vs − 80 °C indicative of sub-optimal storage in general; and second, to define serum metabolites and proteins unaffected by storage at − 20 °C vs − 80 °C.

## Materials and methods

### Participants and serum samples

The Finnish Diabetic Nephropathy (FinnDiane) Study is a prospective nationwide multicenter study comprising more than 8400 adults with type 1 diabetes from 21 university and central hospitals, 33 district hospitals, and 26 primary health care centers across Finland covering more than 10% of all the individuals with type 1 diabetes in Finland^8^. Although, strictly speaking, it is not a population-based study, the geographical distribution of the FinnDiane patients closely follows the distribution of the general population of Finland. Patients participated in the study during a regular visit to their attending physician during which detailed demographic and medical history data were collected with standardized questionnaires.

The Scottish Diabetes Research Network Type 1 Bioresource (SDRNT1BIO)^9^ is a prospective cohort study, comprising 6127 people with a clinical diagnosis of type 1 diabetes, representative of all adults with type 1 diabetes in Scotland, and recruited between December 2010 and November 2013. At recruitment, clinical measurements and a blood sample were taken.

Both studies were performed in accordance with the Declaration of Helsinki; all participants gave their written informed consent, and the study protocol was approved by the local ethics committees (FinnDiane: HUS Helsinki University Hospital, Committee III, ref HUS/3313/2018/6; SDRNT1BIO: Tayside Committee for Research Ethics, Committee B, ref 10/S1402/43).

For this study, we selected a sub-population of 16 FinnDiane individuals, for whom split-aliquot serum samples, stored continuously at − 20 °C and − 80 °C and never thawed, were available. This storage dataset was used as the discovery dataset.

The study was performed as a part of a larger biomarker project which included 315 individuals from the FinnDiane and 916 individuals from the SDRNT1BIO cohorts^10^. The individuals in the larger biomarker project were used to validate biomarkers of storage at − 20 °C vs − 80 °C, as the serum samples from FinnDiane were stored at − 20 °C and the serum samples SDRNT1BIO were stored at − 80 °C. In SDRNT1BIO it took on average 2 h 29 min between when the sample was taken to when it entered the freezers after processing (median time 2 h 15 min, interquartile range: 1 h 35 min–3 h 13 min) and they were not thawed prior to analysis. We do not have data on time from sample collection to freezer for FinnDiane and most of the 315 samples were thawed at least once before analysis.

### Biomarkers

Altogether 300 analytes were measured in non-fasting serum samples using two different targeted platforms: 269 (122 metabolite concentrations and metabolite ratios, and 147 tryptic peptides (relative quantitation)) were measured by liquid chromatography tandem mass spectrometry (LC-MSMS) at the WellChild Laboratory (King's College London, UK) and 31 protein concentrations were measured using the Luminex platform at Myriad RBM (Austin, TX, USA) (Supplementary Table 1).

Several quality control steps were applied to the data before the main analysis. If an analyte had only a single constant value (n = 28) or was missing more than 50% in the 32 measured discovery samples from 16 individuals (n = 8), it was removed from the analysis as uninformative. In addition, 2 analytes failed quantification: ApoD (518.3/824.8) (precursor ion m/z/product ion m/z) due to variable chromatographic separation, while the Tissue Inhibitor of Metalloproteinases 3 (TIMP-3) assay did not meet analytical specifications.

We evaluated the reproducibility of the analyte quantification by measuring duplicate samples (35 duplicate samples in SDRNT1BIO, 25 in FinnDiane) in the pilot phase of the main biomarker project. We calculated intra-class correlation on the duplicate samples in both cohorts separately and removed analytes with intra-class correlation < 0.4 (n = 79) in either cohort.

After removing 107 analytes from the analysis, 193 analytes (27 proteins, 83 metabolites and metabolite ratios, and 83 tryptic peptides) were included. The analyte median (IQR) values or the removal reason in the discovery dataset are listed in Supplementary Table 1.

Left-censored values (Supplementary Table 1 and Ref.^10^) below the detection threshold were imputed to half of the detection threshold and right-censored values (not present in the discovery samples and only present for N-terminal prohormone of brain natriuretic peptide in the validation samples) were imputed to maximum reported value.

### Statistical analysis

To quantify the effect of storage at − 20 °C on the serum analytes we defined a score

$${S}_{d}=\frac{{\mu }_{d}}{{\sigma }_{d}}$$

where ${\mu }_{d}$ is the mean of the difference between analyte levels in − 80 °C and − 20 °C samples for each individual and ${\sigma }_{d}$ the standard deviation of the difference.

Given that samples were paired and within each pair the only difference between samples was the storage temperature, a paired t-test or a paired Wilcoxon signed rank test was performed to quantify the statistical significance of the pairwise difference. The paired Wilcoxon signed rank test was used if the distribution of the pairwise differences did not seem to follow a normal distribution (Shapiro–Wilk test p-value < 0.05), otherwise the paired t-test was utilized.

Further, to determine the effect of storage time on the stability of the analytes a linear model was fitted:

$${\mathrm{log}}_{2}(f{c}_{bm})\sim {t}_{stor}$$

where $f{c}_{bm}$ is the ratio of analyte levels at − 80 °C and − 20 °C (fold change) and ${t}_{stor}$ is the storage time in years. Fold change was used to quantify the effect of storage temperature on the analyte levels to obtain a measure independent of the mean magnitude of the measurement pair. Through the log_2_ transformation, we are modelling the effect of storage time expressed in terms doubling (or halving) of the ratio between analyte levels at the two storage temperatures.

To identify biomarkers predictive of storage temperature, the individual analyte’s ability to discriminate between − 20 °C and − 80 °C samples was evaluated by calculating the receiver operating characteristics (ROC) curve and the corresponding area under the curve (AUC) in the discovery set, using the raw analyte values. In other words, only the raw analyte value was used as the explanatory variable to predict if a sample was stored at − 20 °C or − 80 °C and a ROC curve was constructed using this simple classifier for each potential biomarker. The discriminative power of biomarkers classifying − 20 °C and − 80 °C samples correctly in the discovery set was tested in the combined FinnDiane-SDRNT1BIO validation set. Further, the discovery set was used to find for each biomarker the interval that separates the − 20 °C and − 80 °C samples, such that setting the classification threshold within this interval gives perfect classification in the discovery set. Then ranges of sensitivity and specificity were calculated in the validation set when the classification threshold was within this interval.

### Biomarker panels

We also constructed parsimonious biomarker panels to predict the sample storage temperature using LASSO-penalized regression implemented in the glmnet R-package^11^, where the optimal value of the penalty parameter was learned through internal leave-one-out cross-validation. We first constructed panels separately for the proteins, metabolites and tryptic peptides using the FinnDiane discovery dataset. Based on the historically well-known instability of glutamine in stored serum samples^12^, we also constructed a panel for the metabolites in which glutamate/glutamine ratio was forced into the model. The performance of all panels was tested in the two validation sets by applying the regression coefficients learned on the discovery dataset.

Missing analyte values were imputed to median and analyte values were log_10_-transformed prior to constructing the biomarker panels.

## Results

### Sample characteristics

The median serum sample storage time for the 16 split-aliquot pairs was 4.2 years (1st quartile (Q_1_) = 2.5 years, 3rd quartile (Q_3_) = 7.3 years) and the median storage start year was 2011 (Q_1_ = 2008, Q_3_ = 2013) (Table 1).

**Table 1:** Patient characteristics at the time of sample collection, and storage time of the 16 discovery samples. We report median and interquartile range (IQR) for continuous variables, and N (frequency) for categorical variables.

Covariate	Frequency/median (IQR)
Age (years)	40.0 (31.0, 49.6)
Sex (Female)	1 (6.3%)
Diabetes duration (years)	20.0 (10.1, 30.1)
Length of storage (years)	4.2 (2.5, 7.3)
Start of storage (calendar year)	2011 (2008, 2013)
Analyte measurement (calendar year)	2015 (2015, 2015)

### Analytes affected by storage at − 20 °C vs − 80 °C

**Figure 1:**
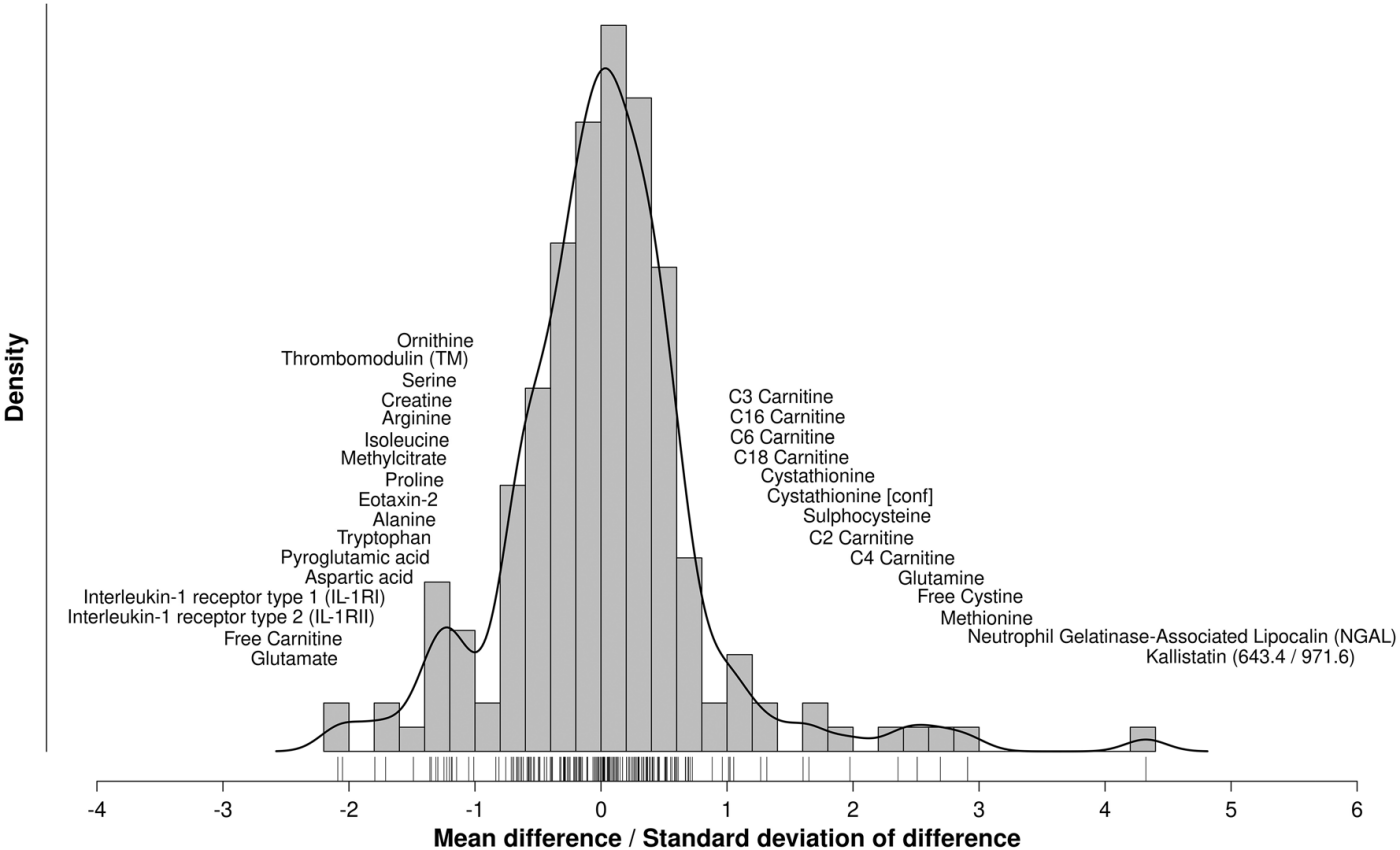
Distribution of mean difference between paired − 80 °C and − 20 °C samples divided by standard deviation of the difference for the analytes in the analysis. Tick marks on the x-axis show the individual data points and the solid line shows the nonparametric density estimator. Analyte names are shown for those with $|{S}_{d}|>1$.

Out of 193 analytes passing quality control (27 proteins, 83 metabolites and metabolite ratios, and 83 tryptic peptides) there were 12 analytes affected by − 20 °C vs. − 80 °C storage temperature, defined as |${S}_{d}|$  > 1.5 (p < 2.6 × 10^–4^ for each analyte, corresponding to a Bonferroni corrected significance threshold of p = 0.05 for multiple testing of 193 analytes). Eight analytes had ${S}_{d}>1.5$, indicating markedly higher concentration when stored at − 80 °C, namely kallistatin (643.4/971.6), neutrophil gelatinase-associated lipocalin, methionine, free cystine, glutamine, C4 carnitine, C2 carnitine and sulphocysteine. Conversely there were four analytes for which ${S}_{d}<-1.5$ (p < 2.6 × 10^–4^) indicating lower concentration when stored at − 80 °C, namely glutamate, free carnitine, interleukin-1 receptor type 2 and interleukin-1 receptor type 1 (Table 2). On the contrary, 120 analytes showed only minimal effect of storage temperature with a $|{S}_{d}|<0.5$ and were defined as robust (Supplementary Table 2). The distribution of ${S}_{d}$ across all analytes is shown in Fig. 1.

**Table 2:** Analytes most affected (cells highlighted with bold) by storage temperature defined as |${\mathrm{S}}_{\mathrm{d}}$| > 1.5 or by storage time in different temperatures defined as storage time associated with ${\mathrm{log}}_{2}\left({\mathrm{fc}}_{\mathrm{bm}}\right)$ at a Bonferroni corrected significance threshold of p < 2.6 × 10^–4^.

Analyte	Storage temperature			Storage time	
	${\upmu }_{\mathrm{d}}$	${\mathrm{S}}_{\mathrm{d}}$	${\mathrm{P}}_{\mathrm{paired}}$	${\mathrm{fc}}_{\mathrm{time}}$	${\mathrm{P}}_{\mathrm{time}}$
Kallistatin (643.4/971.6)	**281.8**	**4.32**	**2.6 × 10**^**–11 t**^	0.90	2.4 × 10^–01^
Neutrophil gelatinase-associated lipocalin	**169.9**	**2.91**	**6.6 × 10**^**–09 t**^	1.06	8.2 × 10^–02^
Methionine	**22.3**	**2.69**	**1.9 × 10**^**–08 t**^	1.29	4.8 × 10^–03^
Free cystine	**55.9**	**2.51**	**3.1 × 10**^**–05 w**^	**1.25**	**1.0 × 10**^**–04**^
Glutamine	**336.8**	**2.36**	**1.1 × 10**^**–07 t**^	**1.31**	**1.8 × 10**^**–04**^
Glutamate	− **201.1**	− **2.09**	**5.0 × 10**^**–07 t**^	0.88	2.7 × 10^–04^
Free carnitine	− **5.3**	− **2.05**	**3.1 × 10**^**–05 w**^	0.99	2.0 × 10^–02^
C4 carnitine	**76.2**	**1.98**	**1.0 × 10**^**–06 t**^	**1.08**	**5.7 × 10**^**–06**^
Interleukin-1 receptor type 2	− **2.1**	− **1.79**	**3.2 × 10**^**–06 t**^	0.99	4.4 × 10^–01^
Interleukin-1 receptor type 1	− **379.6**	− **1.71**	**5.6 × 10**^**–06 t**^	0.93	5.9 × 10^–02^
C2 Carnitine	**4.1**	**1.65**	**8.4 × 10**^**–06 t**^	**1.41**	**4.1 × 10**^**–08**^
Sulphocysteine	**1034.7**	**1.60**	**3.1 × 10**^**–05 w**^	**1.28**	**4.3 × 10**^**–05**^
C6 carnitine	12.6	1.02	4.8 × 10^–04 w^	**1.10**	**1.7 × 10**^**–04**^
C3 CARNITINE	151.2	1.01	4.8 × 10^–04 w^	**1.14**	**7.7 × 10**^**–06**^
Glutamate/glutamine	− 3.4	− 0.63	3.1 × 10^–05 w^	**0.67**	**1.0 × 10**^**–04**^

$f{c}_{bm}$ is the ratio of analyte levels for the paired samples at − 80 °C and − 20 °C, ${\mu }_{d}$ is the mean of the difference between paired − 80 °C and − 20 °C samples, ${S}_{d}$ is the score for difference between paired − 80 °C and − 20 °C samples, ${S}_{d}={\mu }_{d}/{\sigma }_{d}$, where ${\sigma }_{d}$ is the standard deviation of the difference between paired − 80 °C and − 20 °C samples, ${P}_{paired}$ is the paired t-test or Wilcoxon signed rank test p-value for the difference between − 80 and − 20 °C samples, $f{c}_{time}$ is the linear model estimate for the fold change per 1 year of storage, ${P}_{time}$ is the linear model p-value for the $f{c}_{time}$ estimate, ^t^ paired t-test, ^w^ paired Wilcoxon signed rank test.

For eight analytes the storage time was associated with log_2_(fc_bm_) at a Bonferroni corrected significance threshold of p = 0.05/193 = 2.6 × 10^–4^. In other words, the ratio between the paired − 80 °C and − 20 °C samples changed over time for these analytes: C2 carnitine, C4 carnitine, C3 carnitine, sulphocysteine, glutamate/glutamine, free cystine, C6 carnitine and glutamine (Table 2 and Supplementary Fig. 1). Glutamate/glutamine ratio had the biggest change with an estimated fold change of 0.67 per year; for the seven other analytes, the concentrations increased by a fold change ranging from 1.09 to 1.41 per year. To illustrate, for these seven analytes with fold change > 1, the ratio between the paired − 80 °C and − 20 °C samples was higher in the samples that were stored for a longer time compared to the ones stored for a shorter time.

### Biomarkers of storage at − 20 °C vs − 80 °C

**Figure 2:**
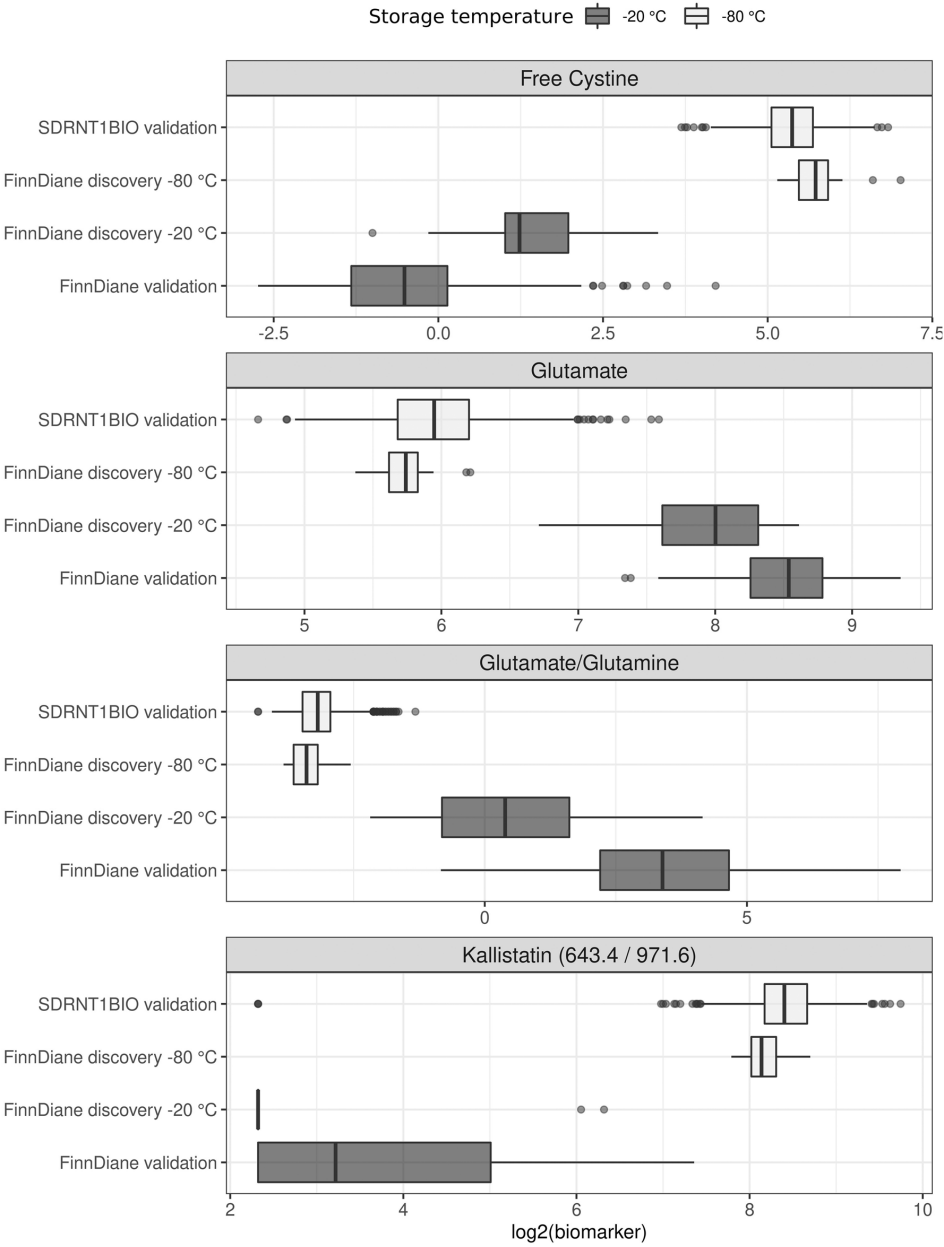
Distribution of log2-transformed values for biomarkers perfectly discriminating between − 20 and − 80 °C samples in the FinnDiane discovery dataset.

The biomarkers discriminating perfectly between samples stored at − 20 °C and − 80 °C in the discovery dataset were free cystine, glutamate, glutamate/glutamine ratio and kallistatin (643.4/971.6). These biomarkers also separated the samples in the validation dataset almost perfectly (AUC > 0.997); glutamate/glutamine ratio had an AUC of 1 (Table 3). The distributions of the log_2_-transformed biomarker values differed also in visual inspection (Fig. 2). The intervals separating the − 20 °C and − 80 °C samples in the discovery dataset are given in Table 4, together with the sensitivity and specificity in the validation dataset, calculated within the separating interval, for each biomarker. Specifically, kallistatin (643.4/971.6) had the most favourable sensitivity range [0.93, 1.00] whereas free cystine had the widest sensitivity range [0.68, 1.00]. The specificity ranges were narrower: specificity was always 1 for glutamate and glutamate/glutamine ratio, [0.99, 1.00] for free cystine and [0.96, 1.00] for kallistatin (643.4/971.6).

**Table 3:** Performance of the candidate biomarkers classifying the samples based on the storage temperature measured by area under the receiver operating characteristics curve (AUC) in the discovery (16 split-aliquot samples stored at − 20 °C and − 80 °C), validation (315 FinnDiane samples stored at − 20 °C and 916 SDRNT1BIO samples stored at − 80 °C) and combined set.

Biomarker	Platform	AUC discovery FinnDiane storage (N = 32)	AUC validation FinnDiane biomarker (N = 315) SDRNT1BIO biomarker (N = 916)	AUC combined All datasets (N = 1263)
Free cystine	LC-MSMS metabolites	1	0.99997	0.99997
Glutamate/glutamine	LC-MSMS metabolites	1	1	0.99988
Glutamate	LC-MSMS metabolites	1	0.99998	0.99965
Kallistatin (643.4/971.6)	LC-MSMS tryptic peptides	1	0.99748	0.99756

**Table 4:** Ranges of candidate biomarkers in the discovery set samples stored at − 20 °C and − 80 °C. Classification interval is the interval which gives perfect classification results in the discovery set. Sensitivity and specificity ranges are given in the validation set when the classification threshold is in the perfect classification interval.

Biomarker	Unit	Range − 20 °C Discovery	Range − 80 °C Discovery	Classification interval Discovery	Sensitivity Validation	Specificity Validation
Free cystine	µM/L	[0.50, 10.10]	[35.40, 129.60]	[10.10, 35.40]	[0.68, 1.00]	[0.99, 1.00]
Glutamate	µM/L	[104.80, 391.30]	[41.40, 74.10]	[74.10, 104.80]	[0.75, 0.95]	[1.00, 1.00]
Glutamate/glutamine	Ratio	[0.22, 17.80]	[0.07, 0.17]	[0.17, 0.22]	[0.90, 0.97]	[1.00, 1.00]
Kallistatin (643.4/971.6)	Ratio	[5.00, 79.80]	[221.20, 416.20]	[79.80, 221.20]	[0.93, 1.00]	[0.96, 1.00]

### Biomarker panels

The four biomarkers for storage at − 20 °C vs. − 80 °C were detected with LC-MSMS metabolite and tryptic peptide platforms. As they may not be readily available in different biomarker studies, we further investigated biomarkers for the storage temperature separately on each biomarker platform. We identified parsimonious biomarker panels separately for proteins, metabolites and tryptic peptides using LASSO-penalized regression (Supplementary Table 3). The panels for each platform consisted of one to five biomarkers and performed well in classifying the samples stored at − 80 °C from the samples stored at − 20 °C: AUC > 0.988 for all panels in the discovery dataset and AUC > 0.995 for all the panels in the validation datasets (Table 5 and Supplementary Table 4).

**Table 5:** Performance of the LASSO-penalised biomarker panels in predicting the storage temperature in the FinnDiane discovery dataset, FinnDiane validation and SDRNT1BIO validation datasets and all datasets combined.

Panel	N biomarkers in the panel/N analytes on the platform	AUC discovery FinnDiane storage (N = 32)	AUC validation FinnDiane biomarker (N = 315) SDRNT1BIO biomarker (N = 916)	AUC combined All datasets (N = 1263)
Luminex proteins	5/27	0.98828	0.99549	0.99536
LC-MSMS metabolites	2/83	1	0.99997	0.99997
LC-MSMS metabolites with glutamate/glutamine	1/83	1	1	0.99990
LC-MSMS tryptic peptides	3/83	1	0.9967	0.99675

## Discussion

Metabolomic and proteomic analyses of patient cohort samples are central to biomarker discovery studies aimed at developing new clinical diagnostics and prognostics. It is generally considered that biomarker discovery is relatively straightforward for acute clinical presentations, as they are usually accompanied by significant protein and/or enzyme release, a characteristic that has been used to develop many of our current repertoire of clinical diagnostics. In addition, rapid outcome measures allow informative samples to be obtained within a relatively short time window and, therefore, should not require long term storage before analysis.

Less straightforward, but arguably clinically more valuable, are the recent initiatives to elucidate new biomarkers that can be used to either define the risk of developing a chronic disease or enable very early detection of evolving disease processes. The aims are to ensure that, in high-risk individuals, early therapeutic intervention ameliorates disease progression, and, in low risk individuals, reassurance is afforded, and clinical intervention minimised. In addition, in the current era, it is anticipated that new clinical biomarkers will aid cost-effective assessment of new therapeutics. Unfortunately, the progression of chronic diseases, e.g. cirrhosis, tends to be slow and the development of clinical complications insidious, e.g. diabetic nephropathy. The implication is that, to identify early diagnostic/prognostic biomarkers in slowly progressing diseases requires the collection and storage of biological samples many years or even decades in advance of the manifestation of easily measurable clinical endpoints. This is particularly true regarding the clinical complications of both type 1 diabetes and type 2 diabetes. In the case of type 1 diabetes the diagnosis is usually made very early in the disease, providing the opportunity to collect biological samples, to establish the natural history of the development of disease complications, both cross-sectionally and longitudinally. If these samples are going to be valuable in future metabolomic and proteomic biomarker studies, it is essential that they are stored appropriately.

On the contrary, many of the studies that collected samples a decade or more ago, stored them at − 20 °C, and the samples may have been subject to multiple freeze–thaw cycles and other sub-optimal pre-analytical factors and storage conditions. To assess the usability of such studies in the current biomarker analyses, we evaluated the effect of long-term storage of serum samples at − 20 °C vs − 80 °C for a total of 193 metabolites and proteins. We identified 15 serum metabolites and proteins that are definitely susceptible to sub-optimal storage, defined as |S_d_| > 1.5, or storage time associated with log_2_(*fc*_*bm*_) (Table 2). Consequently, it could be misleading, if serum samples stored at − 20 °C are used for untargeted biomarker discovery studies, especially, if the stability of the analytes is not known, the serum samples have been stored for variable time periods, or they are combined with samples stored at − 80 °C. On a more positive note, we identified 120 serum metabolites and proteins that appear to be relatively unaffected by storage at − 20 °C vs − 80 °C for up to 7 years (Supplementary Table 2). The implication is that, it would be possible, where samples from a highly informative clinical cohort have been sub-optimally stored, to use the cohort samples in discovery and validation studies, provided the metabolites and/or proteins have been previously demonstrated to be stable.

Furthermore, we defined a serum glutamate/glutamine ratio > 0.20 as being highly sensitive and specific in identifying samples that have been stored at − 20 °C compared to − 80 °C. While we only considered the storage temperature in this analysis (a pre-analytical factor that is often known), we hypothesize that the glutamate/glutamine ratio can be used as a general indicator of sub-optimal storage conditions due to other, unknown pre-analytical factors.

In recent years considerable effort has been expended in defining the effects of sample collection tubes, initial processing before prolonged storage, shipment, storage conditions, and freeze–thaw cycles on metabolomic^12,13^ and proteomic profiles^13^. Studies on the effects of long-term sample storage have focused on − 80 °C ^14,15^. However, there were no internationally recognised sample collection and storage guidelines in place when many of the major clinical cohort studies were originally instigated and, primarily due to cost considerations, many of the samples were stored at − 20 °C. In the case of plasma/serum samples stored continuously at − 20 °C the temperature is insufficient to ensure complete “freezing”, resulting in slow but continued enzymatic conversion of metabolites and protease breakdown of proteins^16–19^. In addition, there are many non-enzymatic processes that continue in samples frozen at − 20 °C, e.g. Schiff base formation, dehydration, hydrolysis, carbamylation, and oxidation^20^, all of which will not only alter specific metabolite concentrations^21^ but also, almost certainly, modify the immunospecificity of certain proteins^22^. Freeze–thaw cycles are even more critical as they provide significant opportunities for further enzymatic and non-enzymatic metabolism. Finally, even in screw-topped tubes there will be significant sublimation of samples, if they are stored for a decade or more at − 20 °C.

Although not investigated in this study, it is appropriate to point out that the temperature of storage of urine samples is even more critical, particularly for proteins: significant losses of all proteins occur in a high proportion of urine samples during initial freezing at − 20 °C ^23^.

A consideration that most biological matrix metabolite and protein stability studies fail to consider is the role of the underlying clinical condition. The majority of stability studies are based on relatively few samples from healthy subjects and the results are then applied to clinical cohort samples on the false assumption that all samples are equal. Plasma enzyme activities vary significantly depending on the disease process being investigated and the underlying sample matrix. In diseases with known extreme phenotypes, e.g. renal disease and uraemia, it is critical that metabolite and protein stability are investigated using appropriate clinical samples. Diabetes is the most obvious extreme phenotype with, at least, the effects of both hyperglycaemia/glycosuria, ease of Schiff base formation with reactive glucose^21^, and increased lysosomal enzyme release^24^, enzymatic conversion and modification, to be considered.

The reasonable concerns over biological sample stability has resulted in significant improvements in current best practice guidelines for optimal collection and storage of plasma/serum samples, including splitting samples into multiple aliquots for single use, to avoid multiple freeze–thaw cycles, and storage at − 80 °C ^2^. The LacaScore has been suggested as a quality control of optimal pre-centrifugation time and temperature of plasma samples^25^. In a similar fashion it would be valuable to define an objective quality control check of optimal prolonged sample storage.

Although we had only a limited number of paired samples to compare storage data at − 20 °C and, consensus best practice, storage at − 80 °C, the differences between certain metabolites were so dramatic that it is relatively easy to establish a criterion for sub-optimal storage. It is interesting to note, though not unexpected, that the serum concentrations of the three sulphur-containing amino acids, free cystine, methionine, and sulphocysteine, are significantly lower when stored at − 20 °C. This is likely to be primarily due to oxidation, though in the case of free cystine the formation of protein linked disulphides could be a major contributory factor^26,27^. The reduced short chain acylcarnitine concentrations are the result of hydrolysis and are, consequently, mirrored by an increase in free carnitine. However, it is the decrease in glutamine and parallel increase in glutamate concentrations that are most informative in determining sub-optimal storage. Glutamine is considered to be relatively stable in aqueous solutions^28^ but at higher temperatures, where losses can be considerable, there is no equivalent increase in glutamate. In this instance it is likely that the product, due to cyclisation, is pyroglutamate^29^. In contrast, it is well recognised within the clinical plasma/serum amino acid analysis community that optimal measurement of glutamine requires fairly quick separation of serum from whole blood and immediate analysis or storage of the serum at − 80 °C ^30^. In addition, the decrease in glutamine is usually associated with a significant increase in glutamate, suggesting that, at least in part, the reaction is enzymatic, with glutaminase being the most likely candidate. The current data are consistent with conversion of glutamine to glutamate and pyroglutamate in serum samples stored at − 20 °C (Supplementary Table 2): the sums of glutamate, glutamine, and pyroglutamate concentrations are equivalent, approximately 600 µmol/L, in the samples under both storage conditions.

The associations between glutamine and glutamate/glutamine ratio with storage time at − 20 °C suggest that this ratio may be used to define varying degrees of sub-optimal storage. In this small dataset, inspection of the serum glutamate/glutamine ratio and simply taking the mean of the highest ratio in the samples stored at − 80 °C and the lowest ratio in those stored at − 20 °C provides a pragmatic discriminatory ratio of 0.20. When applied in the validation dataset the AUC was 1, demonstrating the sensitivity and specificity of this ratio in providing an objective and practical method of determining sub-optimal storage of serum samples. Although this ratio has only been validated in serum samples from individuals with type 1 diabetes, it is highly likely, given the history and our understanding of the conversion process, that it will be applicable to serum samples from disease cohorts other than diabetes. Consequently, we would argue that the glutamate/glutamine ratio should be measured in all serum samples used in metabolomic and proteomic studies as a quality control biomarker of sub-optimal sample storage.

Free cystine and the tryptic peptide of kallistatin (m/z 634.4/971.6) also provide virtually perfect discrimination and, where available, could support the glutamate/glutamine ratio.

Serum retains significant protease and peptidase activities, so it might be expected that storage of samples at − 20 °C would result in significant losses of a number of proteins and tryptic peptides of the 27 proteins and 83 tryptic peptides studied. Interestingly, this was not the case, with only kallistatin (643.4/971.6) and neutrophil gelatinase associated lipocalin (NGAL) particularly susceptible to loss of concentration. In contrast, 14 (52%) proteins and 69 (83%) tryptic peptides were robust against storage temperature. An oblique confirmation of this observation is provided by the metabolite stability data, as any significant proteolytic activity in stored samples would result in increased free amino acid concentrations; this was not the case. Difficult to explain is the apparent increase in the 2 interleukin receptors though this may be due to enzyme activity releasing or exposing more receptor.

To conclude, this study indicates just how vulnerable metabolomic and proteomic cohort studies are to sub-optimal sample storage. We defined a serum glutamate/glutamine ratio > 0.20 as being highly sensitive and specific in identifying samples, from individuals with type 1 diabetes, that have been stored at − 20 °C compared to − 80 °C, and suggest that the ratio can be used as an indicator of sub-optimal storage conditions in general. In addition, we reported 15 serum metabolites and proteins that are highly susceptible to storage at − 20 °C vs − 80 °C and provide an evidence base for the exclusion from untargeted biomarker discovery studies of serum samples stored at − 20 °C. On the contrary, we also identified 120 of the 193 examined serum metabolites and proteins relatively unaffected by sub-optimal storage, supporting that also cohort samples stored at − 20 °C may be used in biomarker studies.

## Supplementary Information

Supplementary Information.

## Data Availability

Summary level data is available in the supplementary information. Individual level data cannot be shared due to patient privacy.
