# Supplementary material for: Effect of serum sample storage temperature on metabolomic and proteomic biomarkers
Source: Sci Rep. 2022 Mar 17;12:4571. doi: 10.1038/s41598-022-08429-0 (PMC8930974; doi:10.1038/s41598-022-08429-0)
Supplement: Supplementary file 1 — Supplementary Information. [file 41598_2022_8429_MOESM1_ESM.docx]

Supplementary Information

Effect of serum sample storage temperature on metabolomic and proteomic biomarkers

Erkka Valo, Marco Colombo, Niina Sandholm, Stuart J. McGurnaghan, Luke A.K. Blackbourn, David B. Dunger, Paul M. McKeigue, Carol Forsblom, Per-Henrik Groop, Helen M. Colhoun, Charles Turner, R. Neil Dalton on behalf of the SDRNT1BIO Investigators and the FinnDiane Study Group

Contents

[Supplementary Methods 3](#_Toc96000372)

[Electrospray MSMS 3](#_Toc96000373)

[Myriad RBM platform for multiplexed whole protein analysis 5](#_Toc96000374)

[References 6](#_Toc96000375)

[Supplementary Table 1 7](#_Toc96000376)

[Supplementary Table 2 17](#_Toc96000377)

[Supplementary Table 3 23](#_Toc96000378)

[Supplementary Table 4 24](#_Toc96000379)

[Supplementary Table 5 25](#_Toc96000380)

[Supplementary Figure 1 28](#_Toc96000381)

# Supplementary Methods

## Electrospray MSMS

Two protocols were used to yield quantitative and semi-quantitative information on serum metabolites (N=122) and serum tryptic peptides (N=147). The WellChild laboratory platforms were originally developed to target biomarkers for inherited metabolic disease diagnosis in childhood and this is reflected in some of the biomarkers available on the panel, such as the amino acids and caeruloplasmin. The metabolite platform was built from a series of individual diagnostic clinical assays for class compounds, e.g. acylcarnitines, or single analytes, e.g., creatine. Consequently, the individual metabolite assays have been rigorously standardised and subject to internal and external quality control (ERNDIM plasma amino acids and special assays). The metabolite assay has been described previously^1,2^, but there have been iterations to the methodology as instrumentation has improved.

The assay is in routine clinical use at the Evelina London Children’s Hospital.

In the main biomarker project^3^, of which this study is a part of, serum samples were analysed in batches of 75. Additional calibrators, aqueous standards at least at 3 concentrations appropriate for normal and pathologically elevated sample concentrations, 2 internal quality control sera, and 3 external quality control sera were also included with each batch.

**Metabolite assay procedure:**

10µL of aqueous standards, controls, and samples were pipetted into 1.8mL polypropylene snap-top Eppendorf tubes. To each tube, 75µL of a stable isotope mixture (free carnitine and acylcarnitines) prepared in methanol was pipetted, followed by 75µL of a second stable isotope mixture (amino acids and other metabolites), also prepared in methanol. The tubes were capped, vortex mixed for 2-5 seconds, and centrifuged at 21,000g at 4°C for 5min. Supernatants, 130µL, were transferred to a 96 deep well (2mL) polypropylene sample block, sealed, and placed in the autosampler at 8°C ready for analysis by LC electrospray MSMS on a Sciex API6500 Q-trap under Analyst 1.6 control. Sample supernatants (3µL) were injected automatically and chromatography performed on an AstecChirobiotic™ T HPLC column 25cm x 2.1mm, 5µm with a 2cm x 4.0mm, 5µm guard column with an isocratic running solvent (acetonitrile:water, 1:1, with 0.025% formic acid) at a flow rate of 200µl/min. Data were acquired in positive ion MRM mode for 7.5min. Once the batch was complete, sample supernatants (10µL) were injected automatically for the acquisition of data for some lower concentration metabolites. Chromatography was performed on an AstecChirobiotic™ T HPLC column 10cm x 2.1mm, 5µm with a 2cm x 4.0mm, 5µm guard column with an isocratic running solvent (acetonitrile:water, 1:1, with 0.025% formic acid) at a flow rate of 225µl/min. Data were acquired in positive ion MRM mode for 10.5min. Once the batch was complete, the sample block was placed in an autosampler at 8°C ready for analysis by LC electrospray MSMS on a Sciex API5000 under Analyst 1.5.2 control. Sample supernatants (10µL) were injected automatically and chromatography performed on an AstecChirobiotic™ T HPLC column 25cm x 2.1mm, 5µm with a 2cm x 4.0mm, 5µm guard column with an isocratic running solvent (acetonitrile:water, 32.5:67.5), at a flow rate of 225µl/min. Data were acquired in negative ion MRM mode for 10min. Quantitation was based on the isotope ratio for each analyte in the sample compared to the isotope ratio in aqueous standards run at the beginning and end of each analytical batch assay. All data were processed in Multiquant 3.0.3. Standard curves, stable isotope ratio vs. concentration, were generated by linear interpolation least squares regression with 1/x weighting; correlation coefficients were typically >0.99, but not <0.97. The analytical integrity of each batch assay was assessed, primarily, using the ERNDIM EQA target values. Batch to batch analytical integrity was assessed using the internal and external QA variability, typically c.5%, but not >15%. Calculated sample metabolite concentrations were exported to Excel. Note that not all metabolites had a corresponding stable isotope internal standard. Where a stable isotope internal standard was not available for a particular compound, a structurally similar stable isotope, already included, was used for quantitation. A total of 73 stable isotope internal standards were included. This not only allows for any losses during sample preparation but also corrects for any ion suppression and changes in MSMS sensitivity.

The tryptic peptide analytical platform is less formally characterised than the metabolite platform, but highly targeted to the more abundant plasma proteins. It is semi-quantitative, all peptides normalised to a single stable isotope peptide, 2H8-albumin T6, which was included in every sample. The method has been published previously^2^.

The assay is in routine clinical use at the Evelina London Children’s Hospital.

**Tryptic peptide assay procedure:**

10µL of Siemens protein standard, neat and diluted 1:5 with deionised water, internal plasma and serum controls, and samples were pipetted as above. To each tube was added, 40µl of water and 50µL of aqueous stable isotope labelled albumin T6 internal standard. The tubes were mixed and 10µL of acetonitrile and 10µL of 1% formic acid added, vortex mixed for 5sec, centrifuged at 10,000g for 5 sec, and mixed on an orbital shaker at RT for 5min. 6µL of 1M NH4CO3 and 25µL of trypsin (5mg/mL) were then added to each tube, the tubes vortex mixed for 5sec, and incubated at 37°C for 1h in an orbital shaker. After incubation, 200µL of running buffer (acetonitrile:water, 1:1, with 0.025% formic acid) was added to each tube, the tubes vortex mixed for 5sec, and centrifuged at 21,000g at 4°C for 5min. Supernatants, 200µL, were transferred to a 96 deep well (2mL) polypropylene sample block, sealed, and placed in the autosampler at 8°C ready for analysis by LC electrospray MSMS on a Sciex API6500 Q-trap under Analyst 1.6 control. Sample supernatants (5µL) were injected automatically and chromatography performed on an AstecChirobiotic™ T HPLC Guard column, 2cm x 4.0mm, 5µm with an isocratic running solvent (acetonitrile:water, 1:1, with 0.025% formic acid) at a flow rate of 600µl/min. Data were acquired in positive ion MRM mode for 10min. All data were processed in Multiquant 3.0.3: only albumin was formally quantified, other peptides being normalised to the albumin T6 stable isotope internal standard. The analytical integrity of each batch assay was assessed using the internal controls. Batch to batch analytical integrity was assessed using the internal QA variability, typically c.5%, but not >15%. Calculated albumin sample metabolite concentrations and peptide ratios were exported to Excel.

It is important to emphasise, though indicated in the main text, that in order to demonstrate the analytical validity of both MSMS platforms, before committing the extremely valuable study samples, a pilot study was performed. 75 serum samples, including multiple duplicates, were provided for “blind” analysis and the results independently scrutinised to remove metabolites and tryptic peptides that did not meet acceptable analytical sensitivity or imprecision criteria. The results presented are only for metabolites or tryptic peptides that met the criteria.

## Myriad RBM platform for multiplexed whole protein analysis

Myriad RBM (MRBM) provide a commercial analytical solution using well described and validated Luminex technology. An additional aliquot of each of the serum samples analysed by MSMS were provided to Myriad RBM for Luminex analysis of proteins that were considered as potentially informative to the primary study objectives. In brief, the company performs multiplexed, microsphere-based assays in a single reaction vessel by combining optical classification schemes, biochemical assays, flow cytometry and advanced digital signal processing hardware and software. Multiplexing is accomplished by assigning each analyte-specific assay a microsphere set labelled with a unique fluorescence signature. To attain distinct microsphere signatures, two fluorescent dyes, red and far red, are mixed in various combinations using various intensity levels of each dye. Each batch or set of microspheres is encoded with a fluorescent signature by impregnating the microspheres with one of these dye combinations. After the encoding process, an assay-specific capture reagent (i.e., antigens, antibodies, receptors, peptides, enzyme substrates, etc.) is conjugated covalently to each unique set of microspheres. Covalent attachment of the capture reagent to the microspheres is achieved with standard carbodiimide chemistry. After optimizing the parameters of each assay separately, Multi-Analyte Profiles are performed by mixing different sets of the microspheres in a single well of a 96- or 384-format microtiter plate. A small sample volume is added to the well and allowed to react with the microspheres. The assay-specific capture reagent on each individual microsphere binds the analyte of interest. A cocktail of assay-specific, biotinylated detecting reagents (e.g., antigens, antibodies, ligands, etc.), is reacted with the microsphere mixture, followed by a streptavidin-labelled fluorescent "reporter" molecule (typically phycoerythrin). Because the microspheres are in suspension, the assay kinetics are near solution-phase. Finally, the multiplex is washed to remove unbound detecting reagents. After washing, the mixture of microspheres is analysed using the Luminex 100/200™ instrument. Similar to a flow cytometer, the instrument uses hydrodynamic focusing to pass the microspheres in single file through two laser beams. As each individual microsphere passes through the excitation beams, it is analysed for size, encoded fluorescence signature and the amount of fluorescence generated in proportion to the analyte. The resulting data stream is interpreted using proprietary data analysis software developed at MRBM. Assays are run in high density multiplexed panels and the Least Detectable Dose (LDD) is determined as the mean +3 standard deviations of 20 blank readings. The LLOQ is determined by the concentration of an analyte where the measurement of analyte demonstrates a coefficient of variation (CV) of 30%. It represents the lowest concentration of analyte that can be measured with a precision better than or equal to 30%. Appropriate dilutions are made to ensure a quantitative measurement within the limits of the assay. An eight (n=8) point standard curve (S1 – S8) is used to obtain quantitative measurements for each sample. Quality Controls (QC's) are run in duplicate along different points of the curve to ensure both accuracy and precision for each analyte.

Note that the Myriad platform was evaluated and validated in a pilot study as described for MSMS.

# References

1. Prentice, P., Turner, C., Wong, M. C. & Dalton, R. N. Stability of metabolites in dried blood spots stored at different temperatures over a 2-year period. *Bioanalysis* **5**, 1507–1514 (2013).

2. Looker, H. C. *et al.* Biomarkers of rapid chronic kidney disease progression in type 2 diabetes. *Kidney International* **88**, 888–896 (2015).

3. Colombo, M. *et al.* Biomarker panels associated with progression of renal disease in type 1 diabetes. *Diabetologia* **62**, 1616–1627 (2019).

# Supplementary Table 1

***Supplementary Table 1.*** Median, interquartile range (IQR) and range of all analytes analysed in the study for the 16 split-aliquot samples in the FinnDiane discovery dataset and the analytes removed from the analysis including the reason for removal.

|  |  | **Discovery -20 °C** | | | **Discovery -80 °C** | | |
| --- | --- | --- | --- | --- | --- | --- | --- |
| **Biomarker** | **Unit** | **Median (IQR)** | **Range** | **Below (%)** | **Median (IQR)** | **Range** | **Below (%)** |
| ***Luminex proteins*** |  |  |  |  |  |  |  |
| Alpha-1-Microglobulin | µg/mL | 18.5 (13.8, 23.5) | 8.5 - 97.0 | 0.0 | 14.5 (13.0, 18.0) | 5.5 - 73.0 | 0.0 |
| Beta-2-Microglobulin | µg/mL | 2.0 (1.8, 2.4) | 1.1 - 18.0 | 0.0 | 1.9 (1.8, 2.6) | 1.2 - 16.0 | 0.0 |
| Calbindin | ng/mL | Poor intra-class correlation |  | 25.0 |  |  | 81.2 |
| CD27 antigen | U/mL | 53.0 (32.5, 73.0) | 18.0 - 1220.0 | 0.0 | 29.0 (16.8, 47.2) | 5.1 - 806.0 | 0.0 |
| Clusterin | µg/mL | Poor intra-class correlation |  | 0.0 |  |  | 0.0 |
| Cystatin-C | ng/mL | 729.0 (664.5, 858.5) | 465.0 - 4340.0 | 0.0 | 752.0 (656.0, 819.2) | 557.0 - 3100.0 | 0.0 |
| Eotaxin-2 | pg/mL | 1595.0 (1105.8, 2902.5) | 593.0 - 4250.0 | 0.0 | 1320.0 (686.8, 2042.5) | 495.0 - 3340.0 | 0.0 |
| Fatty Acid-Binding Protein, heart | ng/mL | Poor intra-class correlation |  | 87.5 |  |  | 87.5 |
| Fibroblast Growth Factor 21 | ng/mL | 0.2 (0.1, 0.3) | 0.1 - 1.9 | 0.0 | 0.3 (0.2, 0.4) | 0.1 - 1.6 | 0.0 |
| Fibroblast Growth Factor 23 | ng/mL | 0.1 (0.0, 0.1) | 0.0 - 7.3 | 31.2 | 0.1 (0.1, 0.2) | 0.0 - 6.9 | 25.0 |
| Growth-Regulated alpha protein | pg/mL | 104.5 (73.0, 164.8) | 18.0 - 342.0 | 0.0 | 101.0 (87.0, 181.0) | 71.0 - 250.0 | 0.0 |
| Insulin-like Growth Factor-Binding Protein 7 | ng/mL | 50.5 (45.2, 58.5) | 23.0 - 131.0 | 0.0 | 47.0 (42.0, 53.8) | 17.0 - 112.0 | 0.0 |
| Interleukin-1 receptor type 1 | pg/mL | 1285.0 (1217.5, 1727.5) | 706.0 - 2730.0 | 0.0 | 1040.0 (888.8, 1425.0) | 202.0 - 2200.0 | 0.0 |
| Interleukin-1 receptor type 2 | ng/mL | 12.0 (10.0, 15.0) | 7.1 - 22.0 | 0.0 | 9.9 (8.9, 12.2) | 5.3 - 19.0 | 0.0 |
| Interleukin-2 receptor alpha | pg/mL | 2330.0 (1895.0, 2460.0) | 1200.0 - 8620.0 | 0.0 | 2185.0 (1922.5, 2362.5) | 1520.0 - 7020.0 | 0.0 |
| Kidney Injury Molecule-1 | ng/mL | 0.0 (0.0, 0.1) | 0.0 - 1.2 | 56.2 | 0.0 (0.0, 0.0) | 0.0 - 1.0 | 56.2 |
| Latency-Associated-Peptide | ng/mL | 12.5 (9.4, 14.0) | 2.6 - 19.0 | 0.0 | 12.0 (9.7, 13.2) | 3.8 - 17.0 | 0.0 |
| Lectin-Like Oxidized LDL Receptor 1 | ng/mL | 0.8 (0.3, 1.4) | 0.3 - 3.0 | 37.5 | 0.5 (0.3, 1.3) | 0.3 - 2.2 | 50.0 |
| N-terminal prohormone of brain natriuretic peptide | pg/mL | 141.0 (46.0, 406.0) | 7.5 - 5370.0 | 12.5 | 288.5 (129.2, 554.0) | 27.0 - 5280.0 | 0.0 |
| Neutrophil Gelatinase-Associated Lipocalin | ng/mL | 139.5 (101.8, 162.2) | 34.0 - 1560.0 | 0.0 | 300.5 (236.8, 385.0) | 149.0 - 1760.0 | 0.0 |
| Osteopontin | ng/mL | 7.3 (4.9, 8.8) | 3.4 - 37.0 | 0.0 | 10.0 (7.8, 12.2) | 5.5 - 22.0 | 0.0 |
| Osteoprotegerin | pM | 4.9 (4.6, 6.5) | 3.8 - 17.0 | 0.0 | 5.5 (5.0, 6.8) | 3.2 - 17.0 | 0.0 |
| Progranulin | ng/mL | 13.0 (11.8, 16.0) | 7.2 - 25.0 | 0.0 | 13.0 (9.7, 15.0) | 5.7 - 18.0 | 0.0 |
| Tamm-Horsfall Urinary Glycoprotein | µg/mL | 0.1 (0.0, 0.1) | 0.0 - 0.1 | 0.0 | 0.1 (0.0, 0.1) | 0.0 - 0.1 | 0.0 |
| Thrombomodulin | ng/mL | 5.4 (4.4, 6.4) | 3.3 - 20.0 | 0.0 | 5.0 (4.1, 5.8) | 3.0 - 18.0 | 0.0 |
| Thymus-Expressed Chemokine | pg/mL | 278.5 (199.8, 411.0) | 134.0 - 519.0 | 0.0 | 324.5 (243.8, 415.5) | 47.5 - 590.0 | 6.2 |
| Tissue Inhibitor of Metalloproteinases 1 | ng/mL | 152.5 (113.8, 199.5) | 65.0 - 226.0 | 0.0 | 147.5 (126.0, 192.2) | 111.0 - 226.0 | 0.0 |
| Tissue Inhibitor of Metalloproteinases 3 | ng/mL | Failed |  | 25.0 |  |  | 0.0 |
| Trefoil Factor 3 | µg/mL | 0.0 (0.0, 0.0) | 0.0 - 1.3 | 0.0 | 0.1 (0.1, 0.1) | 0.0 - 1.6 | 0.0 |
| Tumor Necrosis Factor Receptor I | pg/mL | 1375.0 (1160.0, 1852.5) | 804.0 - 15000.0 | 0.0 | 1340.0 (1162.5, 1890.0) | 803.0 - 13700.0 | 0.0 |
| Vascular Endothelial Growth Factor | pg/mL | 182.0 (99.2, 229.5) | 49.0 - 546.0 | 0.0 | 170.0 (118.0, 275.5) | 21.0 - 743.0 | 6.2 |
| ***LC-MSMS metabolites*** |  |  |  |  |  |  |  |
| 1-Methyl-histidine | µM/L | 4.4 (2.2, 6.2) | 0.5 - 44.1 | 0.0 | 3.8 (1.9, 6.4) | 0.2 - 40.2 | 6.2 |
| 3-hydroxykynurenine | nM/L | Constant values |  | 100.0 |  |  | 100.0 |
| 3-hydroxytyramine HCl | µM/L | Constant values |  | 100.0 |  |  | 100.0 |
| 3-hydroxytyramine HCl [conf] | µM/L | Constant values |  | 100.0 |  |  | 100.0 |
| 3-Methyl-histidine | µM/L | 4.0 (3.6, 5.0) | 2.4 - 26.4 | 0.0 | 3.6 (3.3, 5.0) | 2.2 - 25.2 | 0.0 |
| 3OMD | nM/L | Poor intra-class correlation |  | 0.0 |  |  | 0.0 |
| 5-HIAA | nM/L | Constant values, Poor intra-class correlation |  | 100.0 |  |  | 100.0 |
| 5-hydroxytryptophan | nM/L | Constant values |  | 100.0 |  |  | 100.0 |
| 8OHdG | nM/L | Constant values |  | 100.0 |  |  | 100.0 |
| Adenine | nM/L | Constant values, High missingness |  | 0.0 |  |  | 0.0 |
| Adenine [conf] | nM/L | Constant values, High missingness |  | 0.0 |  |  | 0.0 |
| Adenosine | nM/L | Constant values |  | 100.0 |  |  | 100.0 |
| ADMA | nM/L | 404.5 (383.5, 511.0) | 280.0 - 620.0 | 0.0 | 438.5 (399.0, 514.2) | 338.0 - 634.0 | 0.0 |
| Alanine | µM/L | 419.6 (342.8, 508.3) | 277.1 - 628.6 | 0.0 | 385.9 (327.3, 455.9) | 266.3 - 604.9 | 0.0 |
| Anthranilic acid | nM/L | Constant values |  | 100.0 |  |  | 100.0 |
| Arginine | µM/L | 135.7 (117.0, 154.3) | 98.6 - 232.9 | 0.0 | 95.9 (85.2, 122.0) | 72.2 - 129.7 | 0.0 |
| Argininosuccinic acid | µM/L | Constant values |  | 100.0 |  |  | 100.0 |
| Argininosuccinic anhydride | NA | Constant values |  | 100.0 |  |  | 100.0 |
| Aspartic acid | µM/L | 40.5 (36.3, 42.4) | 30.7 - 75.6 | 0.0 | 19.0 (15.7, 24.0) | 11.9 - 34.5 | 0.0 |
| Beta-Alanine | µM/L | 9.6 (9.0, 10.6) | 6.3 - 11.9 | 0.0 | 9.4 (8.1, 9.8) | 6.5 - 12.2 | 0.0 |
| C10 Carnitine | nM/L | 152.5 (91.0, 200.5) | 66.0 - 281.0 | 0.0 | 185.0 (89.5, 228.2) | 51.0 - 304.0 | 0.0 |
| C10:1 Carnitine | nM/L | 74.0 (61.0, 88.0) | 33.0 - 180.0 | 0.0 | 85.0 (65.2, 102.5) | 30.0 - 212.0 | 0.0 |
| C12 Carnitine | nM/L | 8.0 (4.4, 11.5) | 2.5 - 14.0 | 25.0 | 5.0 (2.5, 7.5) | 2.5 - 14.0 | 37.5 |
| C14 Carnitine | nM/L | 79.5 (64.0, 108.2) | 25.0 - 147.0 | 12.5 | 106.0 (88.2, 115.8) | 55.0 - 239.0 | 0.0 |
| C14:1 Carnitine | nM/L | 219.0 (172.0, 292.5) | 128.0 - 754.0 | 0.0 | 373.0 (314.8, 462.0) | 60.0 - 879.0 | 6.2 |
| C16 Carnitine | nM/L | 302.5 (294.2, 334.0) | 239.0 - 424.0 | 0.0 | 379.0 (339.0, 434.5) | 249.0 - 508.0 | 0.0 |
| C16:1OH Carnitine | nM/L | 10.0 (10.0, 21.8) | 10.0 - 36.0 | 68.8 | 10.0 (10.0, 28.5) | 10.0 - 33.0 | 56.2 |
| C16OH Carnitine | nM/L | 10.0 (10.0, 27.0) | 10.0 - 73.0 | 62.5 | 10.0 (10.0, 10.0) | 10.0 - 43.0 | 81.2 |
| C18 Carnitine | nM/L | 73.0 (64.0, 109.0) | 25.0 - 146.0 | 12.5 | 211.5 (95.5, 249.0) | 25.0 - 325.0 | 12.5 |
| C18OH Carnitine | nM/L | Poor intra-class correlation |  | 37.5 |  |  | 56.2 |
| C2 Carnitine | µM/L | 2.0 (0.5, 4.3) | 0.5 - 11.9 | 43.8 | 5.8 (4.4, 8.1) | 4.1 - 14.5 | 0.0 |
| C3 Carnitine | nM/L | 324.0 (240.0, 407.0) | 82.0 - 939.0 | 0.0 | 428.0 (352.5, 603.0) | 235.0 - 1008.0 | 0.0 |
| C3DC Malonyl/3OHB | nM/L | 113.0 (81.0, 151.2) | 25.0 - 577.0 | 6.2 | 122.0 (96.5, 153.8) | 74.0 - 612.0 | 0.0 |
| C4 Carnitine | nM/L | 130.5 (101.0, 157.5) | 74.0 - 1248.0 | 0.0 | 195.0 (174.8, 238.8) | 141.0 - 1378.0 | 0.0 |
| C4DC Methylmalonyl | nM/L | Poor intra-class correlation |  | 50.0 |  |  | 81.2 |
| C4DC Methylmalonyl/C5OH | nM/L | 102.0 (93.2, 130.0) | 74.0 - 377.0 | 0.0 | 104.5 (94.2, 120.5) | 65.0 - 370.0 | 0.0 |
| C5 Carnitine | nM/L | 141.5 (123.0, 182.2) | 96.0 - 394.0 | 0.0 | 147.0 (134.2, 165.8) | 99.0 - 369.0 | 0.0 |
| C5DC (Glutaryl) Carnitine | nM/L | 119.0 (115.0, 138.0) | 67.0 - 585.0 | 0.0 | 114.0 (101.8, 126.8) | 86.0 - 546.0 | 0.0 |
| C5DC (Glutaryl) Carnitine [conf] | nM/L | 95.5 (78.0, 129.0) | 63.0 - 513.0 | 0.0 | 106.0 (99.8, 127.0) | 82.0 - 404.0 | 0.0 |
| C5OH specific | nM/L | 25.0 (25.0, 59.0) | 25.0 - 155.0 | 62.5 | 25.0 (25.0, 73.5) | 25.0 - 194.0 | 56.2 |
| C6 Carnitine | nM/L | 27.0 (20.0, 40.2) | 5.0 - 68.0 | 6.2 | 38.0 (29.0, 54.5) | 19.0 - 108.0 | 0.0 |
| C6DC (methylglutaryl) Carnitine | nM/L | 70.5 (60.0, 95.8) | 25.0 - 669.0 | 6.2 | 70.5 (57.0, 97.5) | 25.0 - 629.0 | 18.8 |
| C8 Carnitine | nM/L | 111.0 (67.2, 137.2) | 45.0 - 214.0 | 0.0 | 134.0 (75.8, 166.8) | 49.0 - 223.0 | 0.0 |
| Carnosine | nM/L | Poor intra-class correlation |  | 6.2 |  |  | 93.8 |
| Citrate | µM/L | Constant values, High missingness, Poor intra-class correlation |  | 0.0 |  |  | 0.0 |
| Citrate [conf] | µM/L | Constant values, High missingness, Poor intra-class correlation |  | 0.0 |  |  | 0.0 |
| Citrulline | µM/L | 33.8 (29.4, 38.1) | 21.9 - 86.0 | 0.0 | 33.2 (29.1, 38.2) | 21.1 - 84.9 | 0.0 |
| Creatine | µM/L | 18.6 (14.3, 21.6) | 9.9 - 26.6 | 0.0 | 16.6 (12.8, 20.4) | 8.2 - 25.6 | 0.0 |
| Creatinine | µM/L | 60.0 (52.6, 68.8) | 50.5 - 344.8 | 0.0 | 61.6 (53.5, 71.6) | 50.4 - 344.7 | 0.0 |
| Cystathionine | nM/L | 76.0 (48.5, 137.8) | 7.5 - 1320.0 | 6.2 | 116.0 (69.8, 183.0) | 54.0 - 1473.0 | 0.0 |
| Cystathionine [conf] | nM/L | 75.5 (50.8, 133.5) | 12.5 - 1346.0 | 12.5 | 120.0 (67.5, 170.8) | 40.0 - 1462.0 | 0.0 |
| Dihydrothymine | µM/L | Constant values |  | 100.0 |  |  | 100.0 |
| Dihydrouracil | µM/L | Constant values |  | 100.0 |  |  | 100.0 |
| Free Carnitine | µM/L | 40.1 (33.5, 45.8) | 26.6 - 54.2 | 0.0 | 34.6 (30.2, 38.7) | 21.8 - 50.7 | 0.0 |
| Free Cystine | µM/L | 2.4 (2.0, 4.0) | 0.5 - 10.1 | 0.0 | 53.0 (44.5, 60.6) | 35.4 - 129.6 | 0.0 |
| Free homocystine | µM/L | Constant values |  | 100.0 |  |  | 100.0 |
| Free homocystine [conf] | µM/L | Constant values |  | 100.0 |  |  | 100.0 |
| Free Sialic acid | nM/L | 616.0 (533.2, 772.0) | 390.0 - 5253.0 | 0.0 | 607.0 (523.5, 657.8) | 428.0 - 5687.0 | 0.0 |
| Galactose-1-Phosphate | µM/L | 0.2 (0.1, 0.5) | 0.1 - 1.6 | 50.0 | 0.6 (0.4, 0.7) | 0.1 - 1.7 | 12.5 |
| Galactose-1-Phosphate [conf] | µM/L | Constant values, High missingness |  | 0.0 |  |  | 0.0 |
| Glutamate | µM/L | 259.8 (196.1, 318.3) | 104.8 - 391.3 | 0.0 | 53.5 (49.1, 56.8) | 41.4 - 74.1 | 0.0 |
| Glutamate/Glutamine | ratio | 1.3 (0.6, 3.1) | 0.2 - 17.8 | 0.0 | 0.1 (0.1, 0.1) | 0.1 - 0.2 | 0.0 |
| Glutamine | µM/L | 192.8 (108.3, 349.7) | 21.2 - 480.0 | 0.0 | 564.6 (510.9, 596.6) | 425.9 - 682.9 | 0.0 |
| Glycine | µM/L | 264.4 (224.9, 316.7) | 144.4 - 387.4 | 0.0 | 260.8 (216.3, 303.4) | 184.4 - 388.1 | 0.0 |
| Glycine/Valine | ratio | 0.8 (0.8, 1.1) | 0.5 - 2.0 | 0.0 | 0.9 (0.8, 1.1) | 0.6 - 2.1 | 0.0 |
| Guanidinoacetic acid | µM/L | 2.5 (2.0, 3.1) | 1.1 - 4.0 | 0.0 | 2.5 (2.0, 3.0) | 1.1 - 4.1 | 0.0 |
| Hexitol | µM/L | 9.7 (7.4, 23.9) | 5.4 - 59.0 | 0.0 | 9.3 (6.5, 22.0) | 3.4 - 56.2 | 0.0 |
| Histidine | µM/L | 106.5 (104.6, 114.2) | 69.6 - 153.9 | 0.0 | 95.3 (87.2, 101.8) | 75.8 - 108.4 | 0.0 |
| HMMA | nM/L | Constant values |  | 100.0 |  |  | 100.0 |
| Homovanillic acid | nM/L | 598.5 (453.8, 1085.5) | 344.0 - 1489.0 | 0.0 | 444.5 (290.5, 795.0) | 125.0 - 2006.0 | 12.5 |
| Hydroxyproline | µM/L | 14.7 (10.2, 21.2) | 7.9 - 67.2 | 0.0 | 13.5 (10.4, 18.7) | 6.6 - 67.1 | 0.0 |
| Hypoxanthine | µM/L | 6.7 (5.5, 7.8) | 3.0 - 15.1 | 0.0 | 7.2 (6.1, 8.8) | 5.2 - 13.7 | 0.0 |
| Inosine | nM/L | 714.5 (403.8, 1891.0) | 25.0 - 2764.0 | 6.2 | 2006.5 (928.5, 2732.8) | 493.0 - 9742.0 | 0.0 |
| Isoleucine | µM/L | 85.8 (77.5, 98.2) | 54.8 - 138.0 | 0.0 | 82.1 (65.8, 92.2) | 44.6 - 133.2 | 0.0 |
| Kynurenic acid | nM/L | 54.5 (44.0, 67.0) | 28.0 - 465.0 | 0.0 | 48.5 (44.2, 65.5) | 30.0 - 484.0 | 0.0 |
| Kynurenine | µM/L | 2.6 (2.2, 3.4) | 1.2 - 7.7 | 0.0 | 2.6 (2.2, 3.2) | 1.2 - 6.5 | 0.0 |
| Leucine | µM/L | 154.2 (131.0, 195.5) | 79.1 - 250.3 | 0.0 | 137.3 (127.8, 172.2) | 88.0 - 238.7 | 0.0 |
| Leucine/Alanine | NA | 0.4 (0.3, 0.4) | 0.2 - 0.5 | 0.0 | 0.4 (0.4, 0.4) | 0.2 - 0.5 | 0.0 |
| Lysine | µM/L | 201.9 (188.7, 237.8) | 171.9 - 308.5 | 0.0 | 195.2 (185.4, 218.4) | 159.1 - 268.9 | 0.0 |
| Malate | nM/L | Poor intra-class correlation |  | 81.2 |  |  | 50.0 |
| Methionine | µM/L | 4.8 (2.8, 13.0) | 0.5 - 27.5 | 6.2 | 31.6 (23.7, 35.8) | 19.5 - 52.2 | 0.0 |
| Methylcitrate | nM/L | 1128.5 (808.0, 2130.2) | 597.0 - 3340.0 | 0.0 | 797.5 (498.5, 1217.5) | 125.0 - 2765.0 | 6.2 |
| Methymalonic acid | nM/L | 236.0 (186.8, 295.0) | 155.0 - 5025.0 | 0.0 | 185.5 (139.8, 268.5) | 102.0 - 4719.0 | 0.0 |
| N-acetylaspartate | nM/L | 528.0 (448.0, 613.2) | 365.0 - 1277.0 | 0.0 | 538.0 (480.5, 612.2) | 298.0 - 1030.0 | 0.0 |
| N-acetylaspartate [conf] | nM/L | 528.0 (448.0, 613.2) | 365.0 - 1277.0 | 0.0 | 538.0 (480.5, 612.2) | 298.0 - 1030.0 | 0.0 |
| N-acetylglutamine | nM/L | Constant values, High missingness |  | 0.0 |  |  | 0.0 |
| N-acetylglutamine [conf] | nM/L | 175.0 (175.0, 175.0) | 175.0 - 457.0 | 81.2 | 441.0 (175.0, 468.2) | 175.0 - 707.0 | 37.5 |
| NAG | µmol/L/min | 28.4 (24.9, 32.9) | 14.9 - 43.7 | 0.0 | 28.4 (25.6, 31.2) | 18.5 - 47.5 | 0.0 |
| Neopterin | nM/L | 7.5 (7.5, 7.5) | 7.5 - 55.0 | 81.2 | 7.5 (7.5, 7.5) | 7.5 - 40.0 | 81.2 |
| Nitrotyrosine | nM/L | Constant values |  | 100.0 |  |  | 100.0 |
| Ornithine | µM/L | 184.2 (148.2, 204.4) | 96.6 - 288.5 | 0.0 | 126.2 (111.4, 140.1) | 87.4 - 189.2 | 0.0 |
| Orotic | nM/L | Poor intra-class correlation |  | 0.0 |  |  | 0.0 |
| Phenylalanine | µM/L | 73.0 (66.4, 98.1) | 60.1 - 120.2 | 0.0 | 67.7 (64.7, 74.5) | 53.0 - 100.5 | 0.0 |
| Phenylalanine/Tyrosine | ratio | 1.0 (0.9, 1.1) | 0.7 - 1.6 | 0.0 | 0.9 (0.8, 1.0) | 0.7 - 1.6 | 0.0 |
| Phosphoethanolamine | µM/L | Constant values |  | 100.0 |  |  | 100.0 |
| Phosphoserine | µM/L | Poor intra-class correlation |  | 0.0 |  |  | 0.0 |
| Phosphoserine [conf] | µM/L | Constant values, High missingness |  | 0.0 |  |  | 0.0 |
| Proline | µM/L | 256.1 (226.4, 283.4) | 130.2 - 434.8 | 0.0 | 234.5 (199.3, 252.5) | 124.3 - 415.0 | 0.0 |
| Pyridoxal 5prime-phosphate | nM/L | Constant values |  | 100.0 |  |  | 100.0 |
| Pyridoxal 5prime-phosphate [conf] | nM/L | Constant values |  | 100.0 |  |  | 100.0 |
| Pyroglutamic acid | µM/L | 151.5 (104.8, 259.5) | 25.0 - 436.0 | 12.5 | 25.0 (25.0, 25.0) | 25.0 - 25.0 | 100.0 |
| Quinolinic acid | nM/L | Poor intra-class correlation |  | 0.0 |  |  | 0.0 |
| Sarcosine | µM/L | 6.7 (2.5, 8.4) | 2.5 - 11.0 | 31.2 | 2.5 (2.5, 3.2) | 2.5 - 8.6 | 75.0 |
| SDMA | nM/L | 436.0 (411.8, 549.0) | 362.0 - 2839.0 | 0.0 | 464.0 (421.5, 568.2) | 343.0 - 3002.0 | 0.0 |
| SDMA/ADMA | ratio | 1.2 (1.0, 1.5) | 0.7 - 5.5 | 0.0 | 1.1 (0.9, 1.3) | 0.8 - 5.1 | 0.0 |
| Serine | µM/L | 213.0 (191.5, 241.2) | 160.0 - 258.0 | 0.0 | 168.5 (146.2, 184.8) | 112.0 - 223.0 | 0.0 |
| Succinate | µM/L | Poor intra-class correlation |  | 0.0 |  |  | 0.0 |
| Succinylacetone | nM/L | Constant values |  | 100.0 |  |  | 100.0 |
| Sulphocysteine | nM/L | 100.0 (100.0, 100.0) | 100.0 - 425.0 | 87.5 | 1179.0 (559.2, 1557.5) | 391.0 - 2118.0 | 0.0 |
| Taurine | µM/L | 107.5 (89.8, 123.5) | 30.0 - 174.5 | 0.0 | 102.3 (85.0, 122.8) | 52.8 - 178.3 | 0.0 |
| Threonine | µM/L | 127.0 (101.7, 143.7) | 82.5 - 182.5 | 0.0 | 130.6 (101.2, 148.3) | 79.5 - 186.1 | 0.0 |
| Thymine | nM/L | High missingness |  | 6.2 |  |  | 6.2 |
| TMAO | µM/L | 3.8 (2.5, 6.7) | 1.8 - 40.6 | 0.0 | 3.8 (2.4, 6.9) | 1.5 - 42.3 | 0.0 |
| Tryptophan | µM/L | 65.8 (58.3, 74.4) | 32.7 - 91.1 | 0.0 | 64.4 (55.9, 71.6) | 32.0 - 81.2 | 0.0 |
| Tryptophan/Kynurenine | ratio | 26.3 (20.8, 34.5) | 5.6 - 40.0 | 0.0 | 26.0 (21.9, 31.2) | 5.8 - 38.5 | 0.0 |
| Tyrosine | µM/L | 70.3 (62.2, 95.2) | 38.7 - 125.4 | 0.0 | 70.2 (62.0, 91.9) | 38.8 - 124.3 | 0.0 |
| Uracil | nM/L | Poor intra-class correlation |  | 0.0 |  |  | 0.0 |
| Ureidopropionate | nM/L | Constant values |  | 100.0 |  |  | 100.0 |
| Uric acid | µM/L | 307.0 (273.8, 377.8) | 225.0 - 536.0 | 0.0 | 367.5 (283.8, 464.8) | 234.0 - 548.0 | 0.0 |
| Valine | µM/L | 292.1 (263.1, 321.6) | 180.8 - 370.5 | 0.0 | 280.2 (244.7, 324.2) | 184.4 - 376.0 | 0.0 |
| Xanthine | µM/L | 2.0 (1.7, 2.5) | 0.8 - 3.2 | 0.0 | 1.7 (1.6, 1.9) | 1.2 - 2.3 | 0.0 |
| Xanthurenic acid | nM/L | 25.0 (25.0, 63.0) | 25.0 - 131.0 | 62.5 | 25.0 (25.0, 25.0) | 25.0 - 179.0 | 81.2 |
| ***LC-MSMS tryptic peptides (m/z, precursor ion/product ion)*** |  |  |  |  |  |  |  |
| ACTIN (758.9 / 765.4) | * | Poor intra-class correlation |  | 0.0 |  |  | 0.0 |
| Acyloxyacyl hydrolase (703.9 / 931.3) | * | 166.6 (139.4, 195.6) | 108.8 - 231.3 | 0.0 | 143.2 (121.2, 189.2) | 93.8 - 258.8 | 0.0 |
| Afamin (416.8 / 572.4) | * | Poor intra-class correlation |  | 25.0 |  |  | 43.8 |
| Afamin (563.8 / 825.4) | * | Poor intra-class correlation |  | 12.5 |  |  | 12.5 |
| AGR2 (lung cancer biomarker) (407.7 / 701.4) | * | Poor intra-class correlation |  | 0.0 |  |  | 0.0 |
| Alb T31 (337.3 / 416.3) | * | Poor intra-class correlation |  | 0.0 |  |  | 0.0 |
| Alb T34 (441.0 / 680.5) | * | 1113.3 (1065.7, 1126.1) | 983.1 - 1459.0 | 0.0 | 1081.0 (1032.5, 1129.4) | 935.0 - 1250.5 | 0.0 |
| Albumin T6 (575.4 / 937.4) | * | 25359.5 (24523.1, 26448.3) | 22796.6 - 30610.2 | 0.0 | 23970.1 (23514.8, 25180.7) | 19033.8 - 27967.5 | 0.0 |
| Albumin T70 (501.2 / 587.5) | * | 4561.8 (4167.1, 4733.9) | 3835.1 - 5611.9 | 0.0 | 4327.4 (4142.6, 4573.4) | 3837.7 - 5003.2 | 0.0 |
| Alpha-1-acid-glycoprotein 1 (723.3 / 937.4) | * | 92.8 (65.2, 110.8) | 42.5 - 134.3 | 0.0 | 87.7 (62.1, 112.5) | 35.8 - 168.9 | 0.0 |
| Alpha-1-antichymotrypsin iso1 (608.4 / 775.4) | * | 266.5 (237.0, 282.5) | 162.5 - 346.6 | 0.0 | 255.8 (234.3, 269.3) | 196.2 - 440.3 | 0.0 |
| Alpha-1-antitrypsin (444.8 / 718.4) | * | 64.7 (50.5, 76.5) | 41.4 - 105.0 | 0.0 | 74.8 (65.6, 81.2) | 44.6 - 118.1 | 0.0 |
| Alpha-1-antitrypsin (556.4 / 797.7) | * | 41.5 (35.1, 49.3) | 21.7 - 63.0 | 0.0 | 38.5 (31.0, 48.6) | 26.5 - 65.6 | 0.0 |
| Alpha-1-antitrypsin (631.3 / 889.5) | * | 46.9 (5.0, 50.6) | 5.0 - 88.6 | 31.2 | 64.6 (44.2, 71.8) | 5.0 - 82.0 | 6.2 |
| Alpha-1B-glycoprotein (619.3 / 894.5) | * | 74.7 (60.1, 89.0) | 27.3 - 138.6 | 0.0 | 86.4 (73.7, 100.0) | 50.6 - 119.2 | 0.0 |
| Alpha-1B-glycoprotein (687.4 / 960.6) | * | Poor intra-class correlation |  | 0.0 |  |  | 0.0 |
| Alpha-2-macroglobulin (697.8 / 737.4) | * | 157.3 (83.2, 216.2) | 70.0 - 250.7 | 0.0 | 161.1 (142.7, 188.7) | 83.1 - 273.7 | 0.0 |
| Alpha-fetoprotein (490.7 / 833.3) | * | 49.5 (40.6, 60.6) | 5.0 - 64.2 | 12.5 | 55.4 (43.5, 71.0) | 29.0 - 96.0 | 0.0 |
| Aminopeptidase B (547.8 / 680.4) | * | Poor intra-class correlation |  | 0.0 |  |  | 0.0 |
| Angiotensin II (349.8 / 136.1) | * | 983.0 (937.4, 1034.9) | 806.8 - 1188.3 | 0.0 | 969.4 (885.2, 1007.5) | 806.5 - 1083.9 | 0.0 |
| Angiotensinogen (634.9 / 956.6) | * | 33.7 (22.3, 49.9) | 5.0 - 56.1 | 18.8 | 42.1 (33.0, 53.3) | 23.3 - 81.7 | 0.0 |
| ApoA-II-pre (486.8 / 546.4) | * | 65.1 (55.8, 72.3) | 41.1 - 120.9 | 0.0 | 79.3 (71.9, 99.6) | 43.5 - 138.2 | 0.0 |
| ApoA-Q1 (626.8 / 422.2) | * | 141.8 (114.0, 153.8) | 65.5 - 208.7 | 0.0 | 137.2 (119.7, 170.5) | 76.5 - 232.7 | 0.0 |
| ApoA1 (700.8 / 1023.5) | * | 473.6 (422.0, 544.5) | 365.2 - 716.2 | 0.0 | 514.6 (463.9, 601.2) | 357.1 - 787.6 | 0.0 |
| ApoB-100 (655.1 / 975.4) | * | Poor intra-class correlation |  | 0.0 |  |  | 0.0 |
| ApoB-Q1 (640.8 / 838.4) | * | 119.2 (81.8, 153.4) | 54.8 - 213.0 | 0.0 | 131.7 (96.1, 166.6) | 79.8 - 195.3 | 0.0 |
| ApoC-I (516.8 / 466.2) | * | 125.8 (101.5, 139.6) | 81.7 - 215.1 | 0.0 | 160.4 (121.6, 186.8) | 5.0 - 212.7 | 6.2 |
| ApoC-II (745.1 / 1149.7) | * | Poor intra-class correlation |  | 93.8 |  |  | 43.8 |
| ApoC-III (598.8 / 854.4) | * | 202.9 (174.1, 283.1) | 105.9 - 624.2 | 0.0 | 257.6 (219.8, 317.0) | 150.5 - 647.2 | 0.0 |
| ApoD (518.3 / 824.8) | * | Failed |  | 0.0 |  |  | 0.0 |
| ApoE-Q (484.8 / 588.3) | * | 75.3 (54.0, 92.5) | 5.0 - 144.5 | 12.5 | 74.7 (62.5, 83.5) | 43.3 - 151.2 | 0.0 |
| ApoL1 (637.8 / 932.5) | * | 5.0 (5.0, 5.0) | 5.0 - 31.4 | 81.2 | 5.0 (5.0, 31.1) | 5.0 - 58.0 | 56.2 |
| Beta-2-glycoprotein I (511.8 / 751.4) | * | 49.9 (36.2, 58.0) | 5.0 - 106.2 | 6.2 | 43.9 (36.0, 51.9) | 23.9 - 84.9 | 0.0 |
| Beta-2-microglobulin (575.0 / 920.3) | * | 1360.3 (1316.2, 1445.1) | 1120.8 - 1761.9 | 0.0 | 1620.3 (1380.8, 1792.2) | 1178.3 - 1902.4 | 0.0 |
| Bone morphogenetic protein 5 (723.5 / 577.3) | * | Poor intra-class correlation |  | 0.0 |  |  | 0.0 |
| BPI fold-containing family A member 2 (481.3 / 500.4) | * | 2364.0 (2236.4, 2509.1) | 1816.9 - 3062.7 | 0.0 | 2092.5 (1985.5, 2231.8) | 1902.3 - 2514.7 | 0.0 |
| C-C motif chemokine 14 (476.2 / 666.3) | * | Poor intra-class correlation |  | 0.0 |  |  | 0.0 |
| C-reactive protein (564.8 / 609.4) | * | Poor intra-class correlation |  | 0.0 |  |  | 0.0 |
| C-reactive protein (569.7 / 829.3) | * | Poor intra-class correlation |  | 12.5 |  |  | 0.0 |
| C-reactive protein iso1 (696.9 / 1035.6) | * | Poor intra-class correlation |  | 6.2 |  |  | 0.0 |
| C1QTNF1 (547.7 / 707.4) | * | Poor intra-class correlation |  | 0.0 |  |  | 0.0 |
| Caeruloplasmin (602.3 / 695.3) | * | Poor intra-class correlation |  | 0.0 |  |  | 0.0 |
| Caeruloplasmin (760.1 / 1059.8) | * | Poor intra-class correlation |  | 6.2 |  |  | 0.0 |
| Caeruloplasmin (760.6 / 931.4) | * | 62.0 (51.1, 88.7) | 26.1 - 114.3 | 0.0 | 60.9 (54.0, 77.0) | 42.7 - 145.9 | 0.0 |
| Carboxypeptidase A4 (492.9 / 399.3) | * | 193.7 (171.1, 204.1) | 102.8 - 241.8 | 0.0 | 197.2 (178.6, 228.5) | 148.3 - 262.2 | 0.0 |
| Carboxypeptidase M (501.8 / 874.5) | * | Poor intra-class correlation |  | 0.0 |  |  | 0.0 |
| CART std (576.2 / 705.3) | * | 79.5 (59.9, 91.1) | 49.3 - 120.6 | 0.0 | 81.6 (76.8, 89.3) | 49.3 - 114.8 | 0.0 |
| Cathepsin S (359.2 / 717.4) | * | Poor intra-class correlation |  | 18.8 |  |  | 18.8 |
| Cellular repressor of E1A-stimulated genes 1 (575.8 / 704.4) | * | 217.2 (200.7, 244.4) | 156.0 - 264.3 | 0.0 | 218.8 (206.5, 236.4) | 180.9 - 291.3 | 0.0 |
| Cg A (488.2 / 775.4) | * | 28.4 (5.0, 44.4) | 5.0 - 87.6 | 31.2 | 34.2 (5.0, 49.8) | 5.0 - 100.2 | 31.2 |
| Cg B (496.2 / 764.3) | * | 50.9 (42.8, 58.4) | 28.2 - 82.1 | 0.0 | 52.7 (47.0, 64.4) | 36.4 - 95.0 | 0.0 |
| Cg B (579.3 / 815.4) | * | Poor intra-class correlation |  | 0.0 |  |  | 0.0 |
| Chromogranin B (1073.5 / 822.4) | * | Poor intra-class correlation |  | 6.2 |  |  | 6.2 |
| Clusterin (697.5 / 922.4) | * | Poor intra-class correlation |  | 0.0 |  |  | 0.0 |
| Clusterin isoform1 (559.3 / 903.5) | * | 85.1 (65.8, 104.9) | 45.1 - 142.6 | 0.0 | 81.7 (65.5, 103.0) | 47.7 - 145.5 | 0.0 |
| Coagulation factor IX pre (626.3 / 792.4) | * | Poor intra-class correlation |  | 6.2 |  |  | 43.8 |
| Coagulation factor V (555.8 / 898.6) | * | 242.9 (158.9, 305.9) | 5.0 - 418.3 | 12.5 | 259.7 (154.9, 307.9) | 5.0 - 344.2 | 6.2 |
| Coagulation factor V (659.0 / 925.5) | * | Poor intra-class correlation |  | 81.2 |  |  | 12.5 |
| Coagulation factor XIIa HC (442.3 / 685.4) | * | 117.2 (99.8, 144.6) | 67.0 - 171.5 | 0.0 | 95.5 (81.8, 152.4) | 51.4 - 164.0 | 0.0 |
| Complement C1q subcomponent subunit A (420.0 / 244.0) | * | 246.2 (195.9, 294.9) | 145.8 - 361.4 | 0.0 | 264.6 (217.7, 290.0) | 172.5 - 386.3 | 0.0 |
| Complement C3 (501.8 / 731.4) | * | Poor intra-class correlation |  | 0.0 |  |  | 0.0 |
| Complement C3 (673.4 / 646.4) | * | 113.5 (86.3, 132.1) | 76.2 - 174.6 | 0.0 | 123.7 (118.8, 168.3) | 105.0 - 210.7 | 0.0 |
| Complement C4 beta (557.8 / 629.4) | * | 94.7 (75.5, 117.7) | 39.8 - 159.9 | 0.0 | 107.6 (77.8, 125.6) | 35.0 - 136.0 | 0.0 |
| Complement C6 (612.8 / 788.4) | * | Poor intra-class correlation |  | 12.5 |  |  | 18.8 |
| Complement C7 (550.7 / 577.3) | * | Poor intra-class correlation |  | 87.5 |  |  | 87.5 |
| Complement C8 alpha (501.7 / 726.3) | * | Poor intra-class correlation |  | 37.5 |  |  | 18.8 |
| Complement C8 beta (694.9 / 942.6) | * | 416.4 (371.8, 518.1) | 315.8 - 749.8 | 0.0 | 415.2 (380.8, 464.8) | 342.5 - 613.4 | 0.0 |
| Complement C8 beta (809.9 / 1091.5) | * | 76.5 (52.8, 88.2) | 5.0 - 153.9 | 6.2 | 78.3 (56.8, 104.7) | 5.0 - 128.3 | 6.2 |
| Complement C8 gamma (810.9 / 836.4) | * | Poor intra-class correlation |  | 87.5 |  |  | 62.5 |
| Complement factor B (578.3 / 671.4) | * | 234.6 (183.7, 270.2) | 128.0 - 358.6 | 0.0 | 265.8 (210.6, 277.5) | 161.0 - 321.7 | 0.0 |
| Complement Factor I (1007.5 / 1023.5) | * | Poor intra-class correlation |  | 0.0 |  |  | 0.0 |
| Corticosteroid-binding globulin (539.1 / 517.3) | * | Poor intra-class correlation |  | 0.0 |  |  | 0.0 |
| Corticotropin-releasing factor-binding protein (617.8 / 674.3) | * | Poor intra-class correlation |  | 0.0 |  |  | 0.0 |
| Cystatin C (685.0 / 412.1) | * | Poor intra-class correlation |  | 0.0 |  |  | 0.0 |
| E-selectin (613.8 / 982.5) | * | 175.2 (146.9, 202.1) | 94.8 - 256.1 | 0.0 | 188.1 (177.2, 214.0) | 113.7 - 295.3 | 0.0 |
| Extracellular glycoprotein lacritin (481.3 / 501.3) | * | 2091.4 (2008.5, 2139.4) | 1813.6 - 2706.7 | 0.0 | 2117.6 (1925.1, 2181.5) | 1716.2 - 2295.4 | 0.0 |
| Fas (TNFRSF6) associated factor 1 (607.8 / 786.5) | * | 322.4 (285.4, 379.3) | 5.0 - 629.4 | 6.2 | 326.2 (253.4, 397.6) | 5.0 - 769.3 | 6.2 |
| Ferritin HC (673.3 / 1035.5) | * | Poor intra-class correlation |  | 0.0 |  |  | 0.0 |
| Fetuin-B (498.4 / 467.3) | * | Poor intra-class correlation |  | 62.5 |  |  | 68.8 |
| Fibronectin iso1 (536.3 / 680.4) | * | 64.0 (39.9, 115.5) | 5.0 - 156.1 | 6.2 | 82.0 (60.0, 104.5) | 42.2 - 207.6 | 0.0 |
| Fractalkine (612.8 / 977.5) | * | 52.3 (38.0, 63.1) | 5.0 - 123.3 | 6.2 | 62.4 (40.2, 69.9) | 5.0 - 101.3 | 6.2 |
| Haptoglobin (490.5 / 562.6) | * | 135.4 (105.3, 182.2) | 49.4 - 310.9 | 0.0 | 151.4 (128.8, 163.0) | 5.0 - 268.4 | 6.2 |
| Haptoglobin (599.4 / 658.4) | * | 94.8 (86.1, 134.0) | 38.9 - 235.8 | 0.0 | 95.1 (68.3, 110.2) | 29.3 - 245.6 | 0.0 |
| Haptoglobin (602.8 / 803.7) | * | 109.5 (87.8, 128.4) | 5.0 - 189.6 | 6.2 | 101.5 (78.7, 111.0) | 49.4 - 151.3 | 0.0 |
| Haptoglobin beta chain (490.8 / 562.3) | * | Poor intra-class correlation |  | 0.0 |  |  | 0.0 |
| Heat shock protein 60kDa (456.8 / 515.4) | * | 59.0 (52.7, 75.0) | 45.0 - 96.9 | 0.0 | 69.2 (56.9, 78.1) | 39.4 - 95.6 | 0.0 |
| Heat shock protein 90alpha iso1 (675.4 / 921.5) | * | Poor intra-class correlation |  | 93.8 |  |  | 100.0 |
| Hemopexin (571.5 / 650.4) | * | 195.7 (151.2, 231.2) | 108.8 - 310.6 | 0.0 | 204.4 (198.5, 230.8) | 141.7 - 247.8 | 0.0 |
| Hemopexin (610.8 / 959.6) | * | 726.2 (656.0, 793.6) | 495.8 - 1013.9 | 0.0 | 723.2 (667.4, 805.4) | 490.8 - 1006.2 | 0.0 |
| Heparin cofactor II (514.8 / 814.4) | * | 68.2 (57.5, 82.6) | 42.2 - 104.2 | 0.0 | 77.8 (66.8, 84.9) | 50.6 - 98.4 | 0.0 |
| Hyaluronan-binding protein 2 (575.2 / 901.5) | * | 224.7 (213.8, 240.5) | 183.1 - 273.3 | 0.0 | 226.0 (218.7, 238.2) | 190.5 - 249.4 | 0.0 |
| Ig gamma-1 chain C region (594.0 / 698.7) | * | Poor intra-class correlation |  | 6.2 |  |  | 0.0 |
| Immunoglobulin J chain (695.5 / 971.4) | * | 5.0 (5.0, 35.8) | 5.0 - 50.0 | 62.5 | 31.9 (5.0, 59.5) | 5.0 - 75.5 | 43.8 |
| Immunoglobulin lambda-like polypeptide 5 (421.3 / 429.2) | * | 103.0 (96.2, 116.2) | 59.6 - 152.0 | 0.0 | 100.1 (90.1, 118.8) | 58.1 - 146.3 | 0.0 |
| Inhibin beta B chain (594.8 / 419.3) | * | 208.9 (172.1, 238.9) | 101.7 - 258.4 | 0.0 | 179.3 (153.8, 222.2) | 81.1 - 328.2 | 0.0 |
| Insulin-like growth factor-binding protein2 (530.6 / 785.4) | * | Poor intra-class correlation |  | 12.5 |  |  | 37.5 |
| Inter-alpha-trypsin inhibitor HC (579.3 / 902.5) | * | 133.7 (119.9, 141.1) | 97.6 - 180.2 | 0.0 | 138.3 (122.3, 158.6) | 105.0 - 174.4 | 0.0 |
| Interleukin-26 (575.2 / 512.3) | * | 110.5 (102.4, 123.2) | 82.1 - 166.1 | 0.0 | 117.4 (101.1, 137.3) | 90.1 - 162.3 | 0.0 |
| Kallistatin (643.4 / 971.6) | * | 5.0 (5.0, 5.0) | 5.0 - 79.8 | 87.5 | 281.6 (259.6, 317.2) | 221.2 - 416.2 | 0.0 |
| Kininogen-1 (626.3 / 173.1) | * | Poor intra-class correlation |  | 0.0 |  |  | 0.0 |
| Leucine-rich alpha-2-glycoprotein (590.5 / 725.4) | * | 179.0 (167.1, 194.1) | 122.1 - 245.0 | 0.0 | 189.4 (151.6, 209.7) | 117.2 - 224.1 | 0.0 |
| MPO (575.3 / 939.4) | * | Poor intra-class correlation |  | 0.0 |  |  | 0.0 |
| Osteopontin isoA (694.3 / 853.4) | * | 240.8 (209.0, 283.0) | 154.6 - 344.9 | 0.0 | 239.6 (221.9, 272.7) | 165.5 - 363.6 | 0.0 |
| P-selectin (500.2 / 492.3) | * | 270.3 (227.0, 301.9) | 135.5 - 430.6 | 0.0 | 350.8 (275.3, 387.1) | 209.5 - 422.7 | 0.0 |
| P-selectin (712.4 / 876.5) | * | Poor intra-class correlation |  | 68.8 |  |  | 37.5 |
| Pericentriolar material 1 (526.3 / 825.5) | * | 45.5 (41.5, 66.0) | 5.0 - 101.8 | 6.2 | 56.5 (46.6, 68.2) | 5.0 - 87.4 | 6.2 |
| Peroxidase (492.6 / 703.3) | * | 50.9 (37.0, 64.8) | 5.0 - 94.9 | 6.2 | 42.4 (36.2, 50.6) | 23.7 - 85.6 | 0.0 |
| Peroxiredoxin 2 (489.8 / 735.4) | * | 135.2 (128.3, 170.7) | 86.6 - 237.6 | 0.0 | 153.3 (133.2, 168.4) | 75.8 - 215.6 | 0.0 |
| Phosphatidylcholine-sterol acyltransferase (693.7 / 941.5) | * | 99.5 (65.1, 132.7) | 45.2 - 184.2 | 0.0 | 132.3 (99.5, 158.8) | 74.4 - 227.8 | 0.0 |
| Plasma glutamate carboxypeptidase (577.3 / 743.3) | * | Poor intra-class correlation |  | 37.5 |  |  | 43.8 |
| Plasma protease C1 inhibitor (633.0 / 1049.6) | * | Poor intra-class correlation |  | 0.0 |  |  | 6.2 |
| Plasminogen (438.3 / 502.3) | * | Poor intra-class correlation |  | 12.5 |  |  | 12.5 |
| Plasminogen (438.3 / 615.3) | * | 1031.9 (882.0, 1144.8) | 206.5 - 1469.3 | 0.0 | 904.3 (885.9, 956.2) | 745.0 - 1073.9 | 0.0 |
| Plasminogen (570.8 / 699.4) | * | 112.6 (84.6, 127.7) | 58.7 - 148.7 | 0.0 | 104.5 (92.8, 117.4) | 83.8 - 174.3 | 0.0 |
| Polymerase (RNA) II polypeptide D (425.2 / 635.3) | * | 35.0 (31.9, 42.7) | 5.0 - 58.1 | 12.5 | 39.0 (34.8, 49.0) | 5.0 - 64.9 | 6.2 |
| PON1 Serum paraoxonase/arylesterase 2 (942.5 / 472.3) | * | 354.1 (320.3, 415.7) | 136.5 - 564.5 | 0.0 | 350.0 (260.5, 408.3) | 195.0 - 602.2 | 0.0 |
| Prothrombin (626.3 / 679.4) | * | Poor intra-class correlation |  | 12.5 |  |  | 6.2 |
| Prothrombin (626.3 / 879.5) | * | Poor intra-class correlation |  | 0.0 |  |  | 0.0 |
| RBP (583.3 / 669.5) | * | 104.2 (74.3, 122.3) | 33.9 - 290.2 | 0.0 | 122.2 (82.2, 154.4) | 67.4 - 227.9 | 0.0 |
| RBP1 (367.2 / 505.3) | * | Poor intra-class correlation |  | 43.8 |  |  | 50.0 |
| RBP2 (575.8 / 695.3) | * | 4642.9 (4569.2, 4780.0) | 4167.3 - 6013.8 | 0.0 | 4689.4 (4558.1, 5056.6) | 3828.5 - 5721.8 | 0.0 |
| RBP4 (481.2 / 833.4) | * | Poor intra-class correlation |  | 0.0 |  |  | 0.0 |
| Renin isoform1 (520.8 / 502.3) | * | 91.2 (75.0, 111.3) | 44.1 - 201.1 | 0.0 | 77.2 (61.0, 95.2) | 26.5 - 189.6 | 0.0 |
| Renin isoform1 (610.3 / 959.4) | * | Poor intra-class correlation |  | 0.0 |  |  | 0.0 |
| Reticulon-4 isoform1 (618.8 / 894.5) | * | Poor intra-class correlation |  | 0.0 |  |  | 0.0 |
| sCD40L (471.2 / 837.4) | * | Poor intra-class correlation |  | 0.0 |  |  | 0.0 |
| Serotransferrin (489.9 / 735.4) | * | 153.1 (139.3, 193.1) | 107.9 - 255.8 | 0.0 | 178.8 (159.2, 212.6) | 90.3 - 250.4 | 0.0 |
| Serotransferrin (625.5 / 675.4) | * | 306.3 (263.7, 327.6) | 129.9 - 408.3 | 0.0 | 281.5 (227.1, 355.1) | 121.4 - 456.4 | 0.0 |
| Serum Amyloid A (739.4 / 1151.6) | * | Poor intra-class correlation |  | 6.2 |  |  | 12.5 |
| Serum amyloid P-component (578.8 / 508.3) | * | Poor intra-class correlation |  | 0.0 |  |  | 0.0 |
| Somatostatin receptor type 2-P30874 (575.8 / 674.4) | * | 110.9 (104.4, 117.2) | 65.3 - 148.7 | 0.0 | 113.7 (104.0, 121.1) | 62.5 - 151.1 | 0.0 |
| TAO kinase 1 (645.3 / 804.4) | * | 94.8 (85.7, 109.8) | 68.1 - 180.2 | 0.0 | 119.4 (103.6, 128.8) | 53.9 - 184.3 | 0.0 |
| Thyroxine binding globulin (530.8 / 244.1) | * | Poor intra-class correlation |  | 0.0 |  |  | 0.0 |
| Thyroxine-binding globulin (801.1 / 993.9) | * | Poor intra-class correlation |  | 25.0 |  |  | 0.0 |
| TNF alpha (691.9 / 1067.7) | * | Poor intra-class correlation |  | 12.5 |  |  | 0.0 |
| TNFRSF9 (409.7 / 519.3) | * | 333.9 (278.9, 397.6) | 147.7 - 527.5 | 0.0 | 357.2 (224.9, 451.9) | 118.1 - 491.5 | 0.0 |
| Transferrin (489.7 / 735.4) | * | 152.6 (113.2, 181.5) | 105.0 - 217.8 | 0.0 | 154.2 (138.3, 184.9) | 83.7 - 210.3 | 0.0 |
| Transferrin (598.3 / 993.5) | * | 73.6 (61.2, 94.0) | 47.3 - 116.1 | 0.0 | 74.3 (57.3, 86.4) | 5.0 - 100.6 | 6.2 |
| Transferrin (739.9 / 1184.6) | * | Poor intra-class correlation |  | 0.0 |  |  | 0.0 |
| Troponin I (581.8 / 935.4) | * | Poor intra-class correlation |  | 0.0 |  |  | 0.0 |
| Ubiquinol-cytochrome c reductase core protein II (547.3 / 639.3) | * | Poor intra-class correlation |  | 0.0 |  |  | 0.0 |
| Vit D binding (628.2 / 691.3) | * | Poor intra-class correlation |  | 18.8 |  |  | 0.0 |
| Vitamin K-dependent protein C (516.3 / 603.3) | * | Poor intra-class correlation |  | 31.2 |  |  | 12.5 |
| Vitronectin (711.8 / 647.3) | * | 112.5 (94.0, 128.4) | 66.0 - 145.5 | 0.0 | 119.3 (109.9, 138.4) | 86.6 - 151.3 | 0.0 |
| Von Willebrand factor (500.8 / 788.4) | * | 66.5 (62.4, 82.8) | 50.5 - 93.9 | 0.0 | 62.0 (55.4, 69.1) | 41.7 - 90.9 | 0.0 |
| WD repeat domain 67-iso1 (487.8 / 604.3) | * | 49.5 (39.1, 53.2) | 5.0 - 67.3 | 12.5 | 40.6 (17.7, 59.5) | 5.0 - 80.2 | 25.0 |
| Zinc-alpha-2-glycoprotein (697.8 / 789.4) | * | Poor intra-class correlation |  | 25.0 |  |  | 25.0 |

An * specifies that the reported values are the ratio of the biomarker to added synthetic stable isotope labelled albumin T6.
Metabolites with “[conf]” in their names represent confidence transitions.
Below column reports the percentage of observations below the detection threshold.

# Supplementary Table 2

**Supplementary Table 2.** Analytes least affected by storage conditions defined as |$S_{d}$|<0.5.

|  | **Storage temperature** | |  |  | **Storage time** | |  |  |
| --- | --- | --- | --- | --- | --- | --- | --- | --- |
| Analyte | $\boldsymbol{\mu}_{\mathbf{d}}$ | $\boldsymbol{\sigma}_{\mathbf{d}}$ | $\mathbf{S}_{\mathbf{d}}$ | $\mathbf{P}_{\mathbf{paired}}$ | $\mathbf{I}$ | $\mathbf{P}_{\mathbf{I}}$ | $\boldsymbol{\beta}$ | $\mathbf{P}_{\boldsymbol{\beta}}$ |
| ***Luminex proteins*** |  |  |  |  |  |  |  |  |
| Beta-2-Microglobulin | -0.18 | 0.6 | -0.29 | 0.42 ^w^ | -0.04 | 0.68 | 0.0055 | 0.76 |
| Cystatin-C | -83 | 330 | -0.25 | 0.94 ^w^ | 0.069 | 0.54 | -0.016 | 0.44 |
| Fibroblast Growth Factor 21 | 0.014 | 0.14 | 0.099 | 0.13 ^w^ | 0.16 | 0.5 | 0.031 | 0.49 |
| Fibroblast Growth Factor 23 | -0.022 | 0.13 | -0.18 | 1 ^w^ | 0.32 | 0.51 | -0.024 | 0.78 |
| Growth-Regulated alpha protein | -1.4 | 37 | -0.039 | 0.88 ^t^ | -0.31 | 0.37 | 0.094 | 0.14 |
| Interleukin-2 receptor alpha | -150 | 460 | -0.32 | 0.43 ^w^ | -0.0037 | 0.96 | -0.0029 | 0.85 |
| Kidney Injury Molecule-1 | -0.02 | 0.062 | -0.33 | 0.33 ^w^ | -0.053 | 0.86 | -0.0044 | 0.94 |
| Latency-Associated-Peptide | -0.17 | 3.3 | -0.051 | 0.26 ^w^ | -0.37 | 0.28 | 0.084 | 0.18 |
| Osteopontin | 1.4 | 5.1 | 0.27 | 0.065 ^w^ | -0.17 | 0.48 | 0.12 | 0.012 |
| Osteoprotegerin | 0.22 | 0.99 | 0.23 | 0.058 ^w^ | 0.028 | 0.75 | 0.0098 | 0.55 |
| Thymus-Expressed Chemokine | 29 | 87 | 0.33 | 0.2 ^t^ | -0.16 | 0.6 | 0.048 | 0.39 |
| Tissue Inhibitor of Metalloproteinases 1 | 2.6 | 21 | 0.12 | 0.63 ^t^ | -0.015 | 0.91 | 0.016 | 0.53 |
| Tumor Necrosis Factor Receptor I | -52 | 350 | -0.15 | 0.91 ^w^ | -0.035 | 0.36 | 0.0064 | 0.36 |
| Vascular Endothelial Growth Factor | 11 | 89 | 0.13 | 0.62 ^t^ | 0.44 | 0.34 | -0.099 | 0.25 |
| ***LC-MSMS metabolites*** |  |  |  |  |  |  |  |  |
| 1-Methyl-histidine | -0.45 | 1 | -0.43 | 0.038 ^w^ | -0.12 | 0.42 | -0.0067 | 0.81 |
| Beta-Alanine | -0.23 | 1.4 | -0.17 | 0.51 ^t^ | -0.05 | 0.67 | 0.0028 | 0.9 |
| C10 Carnitine | 18 | 40 | 0.45 | 0.22 ^w^ | -0.14 | 0.42 | 0.05 | 0.13 |
| C10:1 Carnitine | 8.2 | 21 | 0.39 | 0.14 ^t^ | -0.082 | 0.65 | 0.038 | 0.26 |
| C16:1OH Carnitine | 2.9 | 13 | 0.22 | 0.4 ^t^ | 0.64 | 0.26 | -0.09 | 0.38 |
| C16OH Carnitine | -5.8 | 20 | -0.28 | 0.4 ^w^ | -0.63 | 0.31 | 0.061 | 0.59 |
| C3DC Malonyl/3OHB | 14 | 33 | 0.42 | 0.11 ^t^ | -0.36 | 0.16 | 0.12 | 0.014 |
| C4DC Methylmalonyl/C5OH | -3.4 | 12 | -0.29 | 0.087 ^w^ | -0.16 | 0.05 | 0.026 | 0.084 |
| C5 Carnitine | 0.38 | 23 | 0.017 | 0.95 ^t^ | -0.21 | 0.04 | 0.049 | 0.011 |
| C5DC (Glutaryl) Carnitine | -4.1 | 25 | -0.16 | 0.52 ^t^ | -0.077 | 0.58 | 0.0057 | 0.82 |
| C5DC (Glutaryl) Carnitine [conf] | -1.1 | 45 | -0.024 | 0.5 ^w^ | 0.16 | 0.44 | -0.0066 | 0.86 |
| C5OH specific | 12 | 61 | 0.2 | 0.43 ^t^ | 0.097 | 0.89 | 0.03 | 0.82 |
| C6DC (methylglutaryl) Carnitine | -7.1 | 18 | -0.4 | 0.13 ^t^ | -0.38 | 0.11 | 0.044 | 0.3 |
| Citrulline | -0.24 | 1.2 | -0.2 | 0.44 ^t^ | -0.055 | 0.033 | 0.0093 | 0.046 |
| Creatinine | 0.67 | 2.6 | 0.26 | 0.31 ^t^ | -0.0061 | 0.81 | 0.0042 | 0.37 |
| Free Sialic acid | -27 | 240 | -0.11 | 0.31 ^w^ | -0.18 | 0.17 | 0.026 | 0.27 |
| Galactose-1-Phosphate | 0.24 | 0.52 | 0.46 | 0.087 ^t^ | 0.87 | 0.27 | -0.0022 | 0.99 |
| Glycine | 3.8 | 43 | 0.088 | 0.73 ^t^ | 0.21 | 0.13 | -0.038 | 0.13 |
| Glycine/Valine | 0.046 | 0.16 | 0.3 | 0.25 ^t^ | 0.26 | 0.086 | -0.041 | 0.13 |
| Guanidinoacetic acid | -0.019 | 0.11 | -0.17 | 0.43 ^w^ | -0.012 | 0.72 | -0.00019 | 0.98 |
| Homovanillic acid | -130 | 270 | -0.49 | 0.039 ^w^ | -0.3 | 0.37 | -0.047 | 0.44 |
| Hypoxanthine | 0.63 | 2.2 | 0.29 | 0.26 ^t^ | 0.029 | 0.91 | 0.029 | 0.54 |
| Kynurenic acid | -1.8 | 8.6 | -0.21 | 0.41 ^t^ | -0.037 | 0.71 | -0.00079 | 0.96 |
| Kynurenine | -0.12 | 0.32 | -0.39 | 0.19 ^w^ | -0.05 | 0.36 | 0.0038 | 0.7 |
| Leucine/Alanine | 0.0019 | 0.034 | 0.055 | 0.32 ^w^ | 0.054 | 0.43 | -0.0087 | 0.48 |
| Methymalonic acid | -47 | 100 | -0.45 | 0.092 ^t^ | 0.055 | 0.75 | -0.072 | 0.039 |
| N-acetylaspartate | 2.4 | 130 | 0.019 | 0.94 ^t^ | -0.014 | 0.94 | 0.0099 | 0.77 |
| N-acetylaspartate [conf] | 2.4 | 130 | 0.019 | 0.94 ^t^ | -0.014 | 0.94 | 0.0099 | 0.77 |
| NAG | 1.5 | 4.5 | 0.34 | 0.19 ^t^ | -0.2 | 0.056 | 0.061 | 0.0036 |
| Neopterin | -2.8 | 9.4 | -0.3 | 0.42 ^w^ | -0.3 | 0.38 | 0.036 | 0.57 |
| SDMA/ADMA | -0.066 | 0.17 | -0.4 | 0.13 ^t^ | -0.014 | 0.88 | -0.0097 | 0.57 |
| Taurine | 0.99 | 17 | 0.057 | 0.82 ^t^ | -0.3 | 0.087 | 0.073 | 0.026 |
| Threonine | 2.6 | 9.2 | 0.28 | 0.28 ^t^ | -0.014 | 0.81 | 0.0077 | 0.47 |
| TMAO | 0.19 | 0.51 | 0.37 | 0.31 ^w^ | 0.043 | 0.37 | -0.011 | 0.22 |
| Tyrosine | -0.88 | 3.6 | -0.25 | 0.34 ^t^ | -0.027 | 0.44 | 0.0024 | 0.7 |
| Xanthine | -0.29 | 0.59 | -0.48 | 0.072 ^t^ | -0.35 | 0.2 | 0.04 | 0.42 |
| Xanthurenic acid | -6.6 | 25 | -0.27 | 0.45 ^w^ | 0.063 | 0.86 | -0.07 | 0.31 |
| ***LC-MSMS tryptic peptides (m/z, precursor ion/product ion)*** |  |  |  |  |  |  |  |  |
| Acyloxyacyl hydrolase (703.9 / 931.3) | -8.4 | 53 | -0.16 | 0.35 ^w^ | -0.065 | 0.79 | -0.0092 | 0.84 |
| Alb T34 (441.0 / 680.5) | -39 | 120 | -0.32 | 0.22 ^t^ | -0.048 | 0.58 | -0.00051 | 0.97 |
| Albumin T70 (501.2 / 587.5) | -150 | 530 | -0.29 | 0.27 ^w^ | -0.1 | 0.27 | 0.012 | 0.48 |
| Alpha-1-acid-glycoprotein 1 (723.3 / 937.4) | 0.52 | 26 | 0.02 | 0.94 ^t^ | 0.058 | 0.8 | -0.025 | 0.56 |
| Alpha-1-antichymotrypsin iso1 (608.4 / 775.4) | 0.75 | 60 | 0.012 | 0.96 ^t^ | -0.059 | 0.72 | 0.013 | 0.66 |
| Alpha-1-antitrypsin (444.8 / 718.4) | 9.2 | 23 | 0.4 | 0.13 ^t^ | 0.43 | 0.077 | -0.048 | 0.26 |
| Alpha-1-antitrypsin (556.4 / 797.7) | -1.2 | 19 | -0.064 | 0.8 ^t^ | -0.2 | 0.56 | 0.032 | 0.61 |
| Alpha-1B-glycoprotein (619.3 / 894.5) | 13 | 32 | 0.41 | 0.12 ^t^ | 0.035 | 0.92 | 0.058 | 0.39 |
| Alpha-2-macroglobulin (697.8 / 737.4) | 14 | 56 | 0.25 | 0.33 ^t^ | -0.067 | 0.81 | 0.054 | 0.29 |
| Alpha-fetoprotein (490.7 / 833.3) | 12 | 30 | 0.41 | 0.12 ^t^ | 0.99 | 0.19 | -0.092 | 0.49 |
| Angiotensin II (349.8 / 136.1) | -20 | 98 | -0.2 | 0.43 ^t^ | -0.023 | 0.78 | -0.00095 | 0.95 |
| ApoA-Q1 (626.8 / 422.2) | 12 | 36 | 0.32 | 0.22 ^t^ | 0.092 | 0.69 | 0.0082 | 0.85 |
| ApoA1 (700.8 / 1023.5) | 44 | 96 | 0.45 | 0.089 ^t^ | -0.031 | 0.82 | 0.032 | 0.22 |
| ApoB-Q1 (640.8 / 838.4) | 9.2 | 34 | 0.27 | 0.3 ^t^ | 0.067 | 0.78 | 0.017 | 0.7 |
| ApoC-I (516.8 / 466.2) | 20 | 43 | 0.46 | 0.083 ^t^ | -0.63 | 0.28 | 0.13 | 0.22 |
| ApoE-Q (484.8 / 588.3) | 8.8 | 33 | 0.27 | 0.29 ^t^ | -0.76 | 0.16 | 0.26 | 0.015 |
| Beta-2-glycoprotein I (511.8 / 751.4) | -4 | 21 | -0.19 | 0.46 ^t^ | 0.26 | 0.61 | -0.048 | 0.61 |
| Caeruloplasmin (760.6 / 931.4) | 3.4 | 26 | 0.13 | 0.62 ^t^ | 0.11 | 0.71 | -0.002 | 0.97 |
| Carboxypeptidase A4 (492.9 / 399.3) | 16 | 42 | 0.37 | 0.16 ^t^ | -0.11 | 0.51 | 0.047 | 0.12 |
| CART std (576.2 / 705.3) | 4.9 | 33 | 0.15 | 0.55 ^t^ | 0.24 | 0.46 | -0.028 | 0.64 |
| Cellular repressor of E1A-stimulated genes 1 (575.8 / 704.4) | 5.6 | 33 | 0.17 | 0.5 ^t^ | 0.077 | 0.5 | -0.0081 | 0.7 |
| Cg A (488.2 / 775.4) | 2 | 31 | 0.064 | 0.8 ^t^ | -0.43 | 0.71 | 0.11 | 0.61 |
| Cg B (496.2 / 764.3) | 3.5 | 20 | 0.17 | 0.51 ^t^ | 0.12 | 0.67 | -0.0046 | 0.93 |
| Clusterin isoform1 (559.3 / 903.5) | -1.4 | 44 | -0.031 | 0.9 ^t^ | -0.47 | 0.2 | 0.097 | 0.16 |
| Coagulation factor V (555.8 / 898.6) | -1.7 | 42 | -0.04 | 0.87 ^t^ | 0.29 | 0.36 | -0.035 | 0.55 |
| Complement C1q subcomponent subunit A (420.0 / 244.0) | 19 | 54 | 0.36 | 0.17 ^t^ | 0.019 | 0.91 | 0.02 | 0.53 |
| Complement C4 beta (557.8 / 629.4) | 3.7 | 27 | 0.14 | 0.63 ^w^ | -0.16 | 0.53 | 0.046 | 0.32 |
| Complement C8 beta (694.9 / 942.6) | -7.7 | 72 | -0.11 | 0.67 ^t^ | -0.12 | 0.3 | 0.023 | 0.26 |
| Complement C8 beta (809.9 / 1091.5) | 1.1 | 41 | 0.026 | 0.92 ^t^ | -0.68 | 0.41 | 0.15 | 0.34 |
| Complement factor B (578.3 / 671.4) | 18 | 59 | 0.3 | 0.26 ^t^ | 0.23 | 0.25 | -0.022 | 0.54 |
| E-selectin (613.8 / 982.5) | 18 | 47 | 0.38 | 0.15 ^t^ | 0.012 | 0.95 | 0.028 | 0.46 |
| Extracellular glycoprotein lacritin (481.3 / 501.3) | -54 | 220 | -0.25 | 0.34 ^t^ | -0.08 | 0.32 | 0.0091 | 0.54 |
| Fas (TNFRSF6) associated factor 1 (607.8 / 786.5) | -1.5 | 73 | -0.021 | 0.93 ^t^ | 0.06 | 0.66 | -0.017 | 0.49 |
| Fibronectin iso1 (536.3 / 680.4) | 18 | 43 | 0.42 | 0.11 ^t^ | 1 | 0.087 | -0.1 | 0.34 |
| Fractalkine (612.8 / 977.5) | 1.4 | 27 | 0.049 | 0.85 ^t^ | -0.24 | 0.51 | 0.053 | 0.43 |
| Haptoglobin (490.5 / 562.6) | -0.87 | 59 | -0.015 | 0.95 ^t^ | -0.35 | 0.52 | 0.04 | 0.68 |
| Haptoglobin (599.4 / 658.4) | -10 | 47 | -0.22 | 0.4 ^t^ | 0.097 | 0.79 | -0.066 | 0.33 |
| Haptoglobin (602.8 / 803.7) | -11 | 37 | -0.29 | 0.27 ^t^ | -0.75 | 0.16 | 0.17 | 0.096 |
| Heat shock protein 60kDa (456.8 / 515.4) | 1.8 | 20 | 0.089 | 0.73 ^t^ | 0.43 | 0.063 | -0.082 | 0.053 |
| Hemopexin (571.5 / 650.4) | 7.6 | 67 | 0.11 | 0.66 ^t^ | -0.0025 | 0.99 | 0.021 | 0.66 |
| Hemopexin (610.8 / 959.6) | -0.41 | 88 | -0.0047 | 0.99 ^t^ | -0.08 | 0.36 | 0.017 | 0.3 |
| Heparin cofactor II (514.8 / 814.4) | 5.8 | 19 | 0.3 | 0.25 ^t^ | 0.055 | 0.8 | 0.019 | 0.64 |
| Hyaluronan-binding protein 2 (575.2 / 901.5) | 0.84 | 34 | 0.024 | 0.92 ^t^ | -0.15 | 0.18 | 0.034 | 0.1 |
| Immunoglobulin lambda-like polypeptide 5 (421.3 / 429.2) | -4.8 | 27 | -0.18 | 0.49 ^t^ | -0.31 | 0.16 | 0.05 | 0.21 |
| Inhibin beta B chain (594.8 / 419.3) | -16 | 63 | -0.26 | 0.32 ^t^ | -0.082 | 0.78 | -0.016 | 0.77 |
| Inter-alpha-trypsin inhibitor HC (579.3 / 902.5) | 7.5 | 21 | 0.36 | 0.17 ^t^ | 0.073 | 0.55 | 0.0017 | 0.94 |
| Interleukin-26 (575.2 / 512.3) | 6.6 | 33 | 0.2 | 0.43 ^t^ | 0.14 | 0.5 | -0.014 | 0.72 |
| Leucine-rich alpha-2-glycoprotein (590.5 / 725.4) | 1.1 | 38 | 0.03 | 0.91 ^t^ | -0.074 | 0.67 | 0.016 | 0.61 |
| Osteopontin isoA (694.3 / 853.4) | 3.7 | 47 | 0.079 | 0.76 ^t^ | -0.1 | 0.51 | 0.027 | 0.34 |
| Pericentriolar material 1 (526.3 / 825.5) | 1.9 | 28 | 0.069 | 0.79 ^t^ | -0.2 | 0.59 | 0.061 | 0.38 |
| Peroxidase (492.6 / 703.3) | -5.2 | 26 | -0.2 | 0.44 ^t^ | -0.86 | 0.063 | 0.18 | 0.039 |
| Peroxiredoxin 2 (489.8 / 735.4) | 0.73 | 32 | 0.023 | 0.93 ^t^ | 0.12 | 0.51 | -0.026 | 0.43 |
| Plasminogen (438.3 / 615.3) | -38 | 360 | -0.11 | 0.25 ^w^ | -0.18 | 0.67 | 0.053 | 0.5 |
| Plasminogen (570.8 / 699.4) | 2.7 | 39 | 0.068 | 0.79 ^t^ | -0.56 | 0.015 | 0.13 | 0.0034 |
| Polymerase (RNA) II polypeptide D (425.2 / 635.3) | 5.5 | 18 | 0.3 | 0.24 ^t^ | -0.94 | 0.13 | 0.26 | 0.029 |
| PON1 Serum paraoxonase/arylesterase 2 (942.5 / 472.3) | -3.5 | 66 | -0.053 | 0.84 ^t^ | -0.062 | 0.71 | 0.013 | 0.66 |
| RBP (583.3 / 669.5) | 13 | 42 | 0.3 | 0.25 ^t^ | -0.035 | 0.9 | 0.06 | 0.26 |
| RBP2 (575.8 / 695.3) | 31 | 490 | 0.063 | 0.8 ^t^ | -0.071 | 0.34 | 0.017 | 0.23 |
| Renin isoform1 (520.8 / 502.3) | -16 | 33 | -0.5 | 0.065 ^t^ | -0.12 | 0.72 | -0.032 | 0.59 |
| Serotransferrin (489.9 / 735.4) | 12 | 50 | 0.24 | 0.35 ^t^ | -0.093 | 0.68 | 0.038 | 0.36 |
| Serotransferrin (625.5 / 675.4) | -9.2 | 86 | -0.11 | 0.67 ^t^ | 0.1 | 0.7 | -0.038 | 0.44 |
| Somatostatin receptor type 2-P30874 (575.8 / 674.4) | 0.05 | 22 | 0.0023 | 0.99 ^t^ | -0.13 | 0.39 | 0.027 | 0.33 |
| TAO kinase 1 (645.3 / 804.4) | 13 | 36 | 0.37 | 0.16 ^t^ | 0.039 | 0.87 | 0.028 | 0.53 |
| TNFRSF9 (409.7 / 519.3) | -0.72 | 190 | -0.0038 | 0.99 ^t^ | 0.4 | 0.43 | -0.092 | 0.32 |
| Transferrin (489.7 / 735.4) | 3.4 | 33 | 0.1 | 0.68 ^t^ | 0.046 | 0.8 | -0.0032 | 0.92 |
| Transferrin (598.3 / 993.5) | -8 | 27 | -0.3 | 0.26 ^t^ | 0.3 | 0.57 | -0.13 | 0.2 |
| Vitronectin (711.8 / 647.3) | 12 | 27 | 0.46 | 0.088 ^t^ | 0.031 | 0.88 | 0.029 | 0.43 |
| Von Willebrand factor (500.8 / 788.4) | -6.9 | 18 | -0.39 | 0.14 ^t^ | -0.073 | 0.72 | -0.018 | 0.64 |
| WD repeat domain 67-iso1 (487.8 / 604.3) | -4.5 | 30 | -0.15 | 0.56 ^t^ | 0.11 | 0.91 | -0.11 | 0.5 |

$\mu_{d}$ is the mean of the difference between paired -80 °C and -20 °C samples,
$\sigma_{d}$ is the standard deviation of the difference between paired -80 °C and -20 °C samples,
$S_{d}$ is $\mu_{d}$ divided by $\sigma_{d}$,
$P_{paired}$ is the paired t-test or Wilcoxon signed rank test p-value for the difference between -80 °C and -20 °C samples,
$I$ is the linear model estimate of the intercept,
$P_{I}$ is the linear model p-value of the intercept estimate,
$\beta$ is the linear model estimate for the storage time effect, and
$P_{\beta}$ is the linear model p-value of the storage time estimate
^t^ paired t-test,
^w^ paired Wilcoxon signed rank test.

# Supplementary Table 3

**Supplementary Table 3.** Biomarker panels produced by LASSO-penalised regression models for prediction of storage temperature, where each panel was generated considering only analytes measured on a single platform. Reported are the selected biomarkers and their unstandardized coefficients per unit of log_10_-transformed biomarker value.

| **Biomarker** | **Beta** |
| --- | --- |
| ***Luminex proteins*** |  |
| Neutrophil Gelatinase-Associated Lipocalin (NGAL) | 7.97 |
| Intercept | -6.90 |
| Lectin-Like Oxidized LDL Receptor 1 (LOX-1) | -4.04 |
| CD27 antigen (CD27) | -3.76 |
| Eotaxin-2 | -1.26 |
| Alpha-1-Microglobulin (A1Micro) | -0.43 |
| ***LC-MSMS metabolites*** |  |
| Intercept | -10.16 |
| Free Cystine | 9.23 |
| Methymalonic acid | -0.51 |
| ***LC-MSMS metabolites with Glutamate/Glutamine*** |  |
| Glutamate/Glutamine | -39.98 |
| Intercept | 4.11 |
| ***LC-MSMS tryptic peptides*** |  |
| Intercept | -15.27 |
| Kallistatin (643.4 / 971.6) | 6.70 |
| Plasminogen (438.3 / 615.3) | 0.49 |
| Angiotensinogen (634.9 / 956.6) | 0.49 |

# Supplementary Table 4

**Supplementary Table 4.** Accuracy and log-likelihood of the biomarker panel models predicting the storage temperature for FinnDiane discovery, FinnDiane validation and SDRNT1BIO validation datasets.

|  | **Discovery** | | **Validation** | | | |
| --- | --- | --- | --- | --- | --- | --- |
| **Panel** | **FinnDiane (N=32) ACC** | **l** | **FinnDiane (N=315) ACC** | **l** | **SDRNT1BIO (N=916) ACC** | **l** |
| Luminex proteins | 0.969 | -6.5 | 0.987 | -25.4 | 0.955 | -198.0 |
| LC-MSMS metabolites | 1.000 | -0.4 | 0.997 | -1.5 | 0.995 | -57.1 |
| LC-MSMS metabolites with Glutamate/Glutamine | 0.969 | -3.2 | 1.000 | 0.0 | 0.987 | -125.8 |
| LC-MSMS tryptic peptides | 1.000 | -1.1 | 0.971 | -23.3 | 0.997 | -62.1 |

ACC=accuracy,
l=log-likelihood

**Supplementary Table 5**

**Supplementary Table 5.** Physicians and nurses at each of the FinnDiane centers participating in patient recruitment and characterization

| **The Finnish Diabetic Nephropathy Study Center** | **Physicians and nurses** |
| --- | --- |
| Anjalankoski Health Center | S. Koivula, T. Uggeldahl |
| Central Finland Central Hospital, Jyväskylä | T. Forslund, A. Halonen, A. Koistinen, P. Koskiaho, M. Laukkanen, J. Saltevo, M. Tiihonen |
| Central Hospital of Åland Islands, Mariehamn | M. Forsen, H. Granlund, A-C. Jonsson, B. Nyroos |
| Central Hospital of Kanta-Häme, Hämeenlinna | P. Kinnunen, A. Orvola, T. Salonen, A. Vähänen |
| Central Hospital of Kymenlaakso, Kotka | R. Paldanius, M. Riihelä, L. Ryysy |
| Central Hospital of Länsi-Pohja, Kemi | H. Laukkanen, P. Nyländen, A. Sademies |
| Central Ostrabothnian Hospital District, Kokkola | S. Anderson, B. Asplund, U. Byskata, P. Liedes, M. Kuusela, T. Virkkala |
| City of Espoo Health Center |  |
| Espoonlahti | A. Nikkola, E. Ritola |
| Tapiola | M. Niska, H. Saarinen |
| Samaria | E. Oukko-Ruponen, T. Virtanen |
| Viherlaakso | A. Lyytinen |
| City of Helsinki Health Center |  |
| Puistola | H. Kari, T. Simonen |
| Suutarila | A. Kaprio, J. Kärkkäinen, B. Rantaeskola |
| Töölö | P. Kääriäinen, J. Haaga, A-L. Pietiläinen |
| City of Hyvinkää Health Center | S. Klemetti, T. Nyandoto, E. Rontu, S. Satuli-Autere |
| City of Vantaa Health Center |  |
| Korso | R. Toivonen, H. Virtanen |
| Länsimäki | R. Ahonen, M. Ivaska-Suomela, A. Jauhiainen |
| Martinlaakso | M. Laine, T. Pellonpää, R. Puranen |
| Myyrmäki | A. Airas, J. Laakso, K. Rautavaara |
| Rekola | M. Erola, E. Jatkola |
| Tikkurila | R. Lönnblad, A. Malm, J. Mäkelä, E. Rautamo |
| Heinola Health Center | P. Hentunen, J. Lagerstam |
| Helsinki University Central Hospital, Department of Medicine, Division of Nephrology | M. Feodoroff, D. Gordin, O. Heikkilä, K. Hietala, J. Fagerudd, M. Korolainen, L. Kyllönen, J. Kytö, S. Lindh, K. Pettersson-Fernholm, M. Rosengård-Bärlund, A. Sandelin, L. Thorn, J. Tuomikangas, T. Vesisenaho, J. Wadén |
| Herttoniemi Hospital, Helsinki | V. Sipilä |
| Hospital of Lounais-Häme, Forssa | T. Kalliomäki, J. Koskelainen, R. Nikkanen, N. Savolainen, H. Sulonen, E. Valtonen |
| Hyvinkää Hospital | L. Norvio, A. Hämäläinen |
| Iisalmi Hospital | E. Toivanen |
| Jokilaakso Hospital, Jämsä | A. Parta, I. Pirttiniemi |
| Jorvi Hospital, Helsinki University Central Hospital | S. Aranko, S. Ervasti, R. Kauppinen-Mäkelin, A. Kuusisto, T. Leppälä, K. Nikkilä, L. Pekkonen |
| Jyväskylä Health Center, Kyllö | K. Nuorva, M. Tiihonen |
| Kainuu Central Hospital, Kajaani | S. Jokelainen, K. Kananen, M. Karjalainen, P. Kemppainen, A-M. Mankinen, A. Reponen, M. Sankari |
| Kerava Health Center | H. Stuckey, P. Suominen |
| Kirkkonummi Health Center | A. Lappalainen, M. Liimatainen, J. Santaholma |
| Kivelä Hospital, Helsinki | A. Aimolahti, E. Huovinen |
| Koskela Hospital, Helsinki | V. Ilkka, M. Lehtimäki |
| Kotka Health Center | E. Pälikkö-Kontinen, A. Vanhanen |
| Kouvola Health Center | E. Koskinen, T. Siitonen |
| Kuopio University Hospital | E. Huttunen, R. Ikäheimo, P. Karhapää, P. Kekäläinen, M. Laakso, T. Lakka, E. Lampainen, L. Moilanen, S. Tanskanen, L. Niskanen, U. Tuovinen, I. Vauhkonen, E. Voutilainen |
| Kuusamo Health Center | T. Kääriäinen, E. Isopoussu |
| Kuusankoski Hospital | E. Kilkki, I. Koskinen, L. Riihelä |
| Laakso Hospital, Helsinki | T. Meriläinen, P. Poukka, R. Savolainen, N. Uhlenius |
| Lahti City Hospital | A. Mäkelä, M. Tanner |
| Lapland Central Hospital, Rovaniemi | L. Hyvärinen, K. Lampela, S. Pöykkö, T. Rompasaari, S. Severinkangas, T. Tulokas |
| Lappeenranta Health Center | P. Erola, L. Härkönen, P. Linkola, T. Pekkanen, I. Pulli, E. Repo |
| Lohja Hospital | T. Granlund, K. Hietanen, M. Porrassalmi, M. Saari, T. Salonen, M. Tiikkainen, |
| Loimaa Health Center | A. Mäkelä, P. Eloranta |
| Länsi-Uusimaa Hospital, Tammisaari | I-M. Jousmaa, J. Rinne |
| Malmi Hospital, Helsinki | H. Lanki, S. Moilanen, M. Tilly-Kiesi |
| Mikkeli Central Hospital | A. Gynther, R. Manninen, P. Nironen, M. Salminen, T. Vänttinen |
| Mänttä Regional Hospital | I. Pirttiniemi, A-M. Hänninen |
| North Karelian Hospital, Joensuu | U-M. Henttula, P. Kekäläinen, M. Pietarinen, A. Rissanen, M. Voutilainen |
| Nurmijärvi Health Center | A. Burgos, K. Urtamo |
| Oulaskangas Hospital, Oulainen | E. Jokelainen, P-L. Jylkkä, E. Kaarlela, J. Vuolaspuro |
| Oulu Health Center | L. Hiltunen, R. Häkkinen, S. Keinänen-Kiukaanniemi |
| Oulu University Hospital | R. Ikäheimo |
| Päijät-Häme Central Hospital | H. Haapamäki, A. Helanterä, S. Hämäläinen, V. Ilvesmäki, H. Miettinen |
| Palokka Health Center | P. Sopanen, L. Welling |
| Pieksämäki Hospital | V. Sevtsenko, M. Tamminen |
| Pietarsaari Hospital | M-L. Holmbäck, B. Isomaa, L. Sarelin |
| Pori City Hospital | P. Ahonen, P. Merisalo, E. Muurinen, K. Sävelä |
| Porvoo Hospital | M. Kallio, B. Rask, S. Rämö |
| Raahe Hospital | A. Holma, M. Honkala, A. Tuomivaara, R. Vainionpää |
| Rauma Hospital | K. Laine, K. Saarinen, T. Salminen |
| Riihimäki Hospital | P. Aalto, E. Immonen, L. Juurinen |
| Salo Hospital | A. Alanko, J. Lapinleimu, P. Rautio, M. Virtanen |
| Satakunta Central Hospital, Pori | M. Asola, M. Juhola, P. Kunelius, M-L. Lahdenmäki, P. Pääkkönen, M. Rautavirta |
| Savonlinna Central Hospital | T. Pulli, P. Sallinen, M. Taskinen, E. Tolvanen, T. Tuominen, H. Valtonen, A. Vartia, S-L. Viitanen |
| Seinäjoki Central Hospital | O. Antila, E. Korpi-Hyövälti, T. Latvala, E. Leijala, T. Leikkari, M. Punkari, N. Rantamäki, H. Vähävuori |
| South Karelia Central Hospital, Lappeenranta | T. Ensala, E. Hussi, R. Härkönen, U. Nyholm, J. Toivanen |
| Tampere Health Center | A. Vaden, P. Alarotu, E. Kujansuu, H. Kirkkopelto-Jokinen, M. Helin, S. Gummerus, L. Calonius, T. Niskanen, T. Kaitala, T. Vatanen |
| Tampere University Hospital | P. Hannula, I. Ala-Houhala, R. Kannisto, T. Kuningas, P. Lampinen, M. Määttä, H. Oksala, T. Oksanen, A. Putila, H. Saha, K. Salonen, H. Tauriainen, S. Tulokas |
| Tiirismaa Health Center, Hollola | T. Kivelä, L. Petlin, L. Savolainen |
| Turku Health Center | A. Artukka, I. Hämäläinen, L. Lehtinen, E. Pyysalo, H. Virtamo, M. Viinikkala, M. Vähätalo |
| Turku University Central Hospital | K. Breitholz, R. Eskola, K. Metsärinne, U. Pietilä, P. Saarinen, R. Tuominen, S. Äyräpää |
| Vaajakoski Health Center | K. Mäkinen, P. Sopanen |
| Valkeakoski Regional Hospital | S. Ojanen, E. Valtonen, H. Ylönen, M. Rautiainen, T. Immonen |
| Vammala Regional Hospital | I. Isomäki, R. Kroneld, L. Mustaniemi, M. Tapiolinna-Mäkelä |
| Vaasa Central Hospital | S. Bergkulla, U. Hautamäki, V-A. Myllyniemi, I. Rusk |

# Supplementary Figure 1
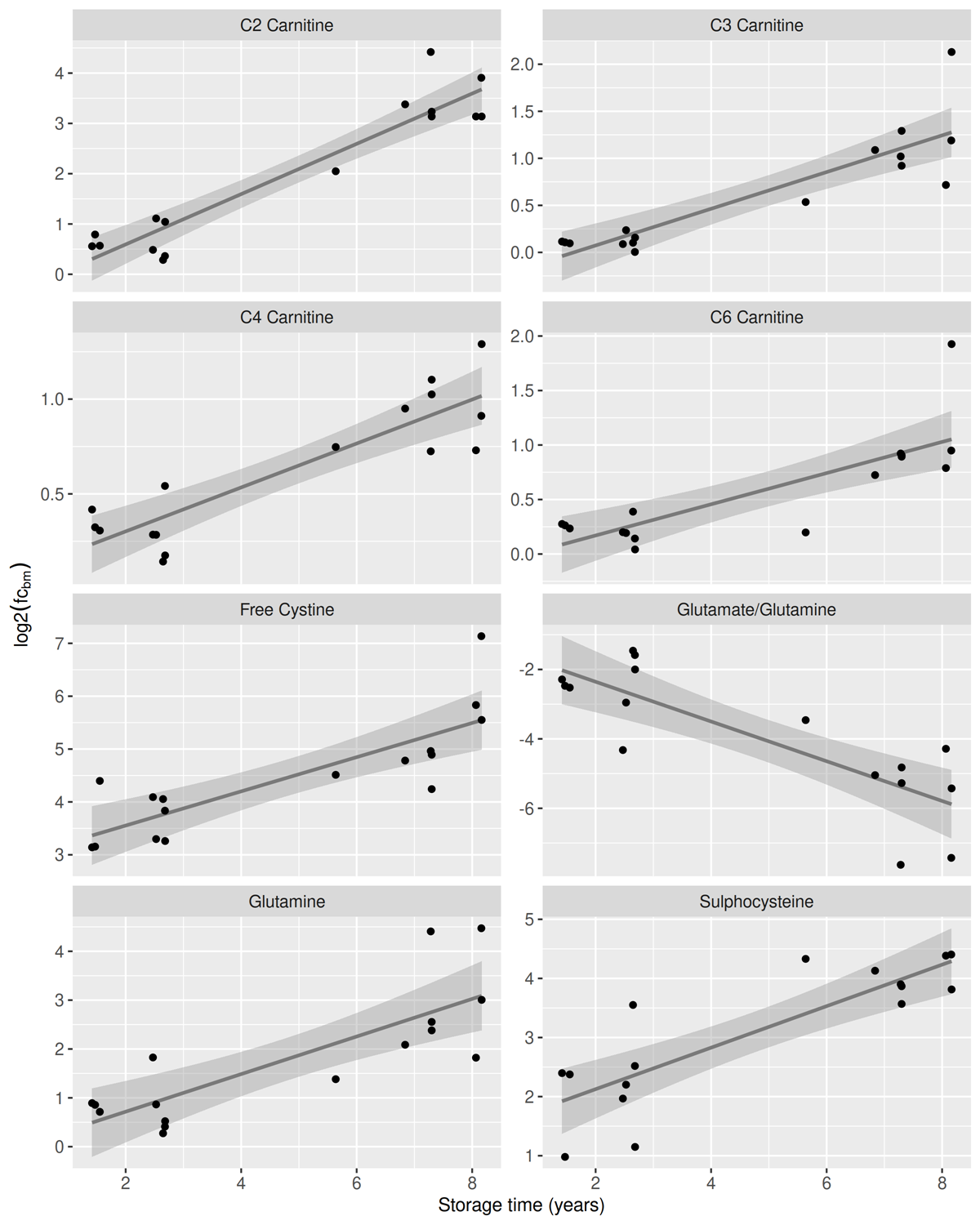


**Supplementary Figure 1.** The effect of storage time for the analytes for which the storage time was associated with log_2_(fc_bm_) at a Bonferroni corrected significance threshold of p = 0.05 /193 = 0.00026.
